# Metastasis suppressing properties of the cell-surface anchored serine protease prostasin: new functional and mechanistic insights from breast cancer

**DOI:** 10.1038/s41389-026-00615-3

**Published:** 2026-04-17

**Authors:** Joseph G. Lundgren, Michael G. Flynn, Ani R. Winkler, Brianna N. Rivera, Lauren M. Tanabe, Paul M. Stemmer, Li-Mei Chen, Karl X. Chai, Karin List

**Affiliations:** 1 Department of Pharmacology, Wayne State University School of Medicine, Detroit, MI USA; 2 Department of Oncology, Wayne State University School of Medicine, Detroit, MI USA; 3 Institute of Environmental Health Sciences, Wayne State University School of Medicine, Detroit, MI USA; 4 Division of Cancer Research, Burnett School of Biomedical Sciences, University of Central Florida College of Medicine, Orlando, FL USA

**Keywords:** Proteolysis, Breast cancer

## Abstract

Serine proteases play multifaceted roles in cancer, affecting tumor formation, progression, and metastasis. While most serine proteases studied act as tumor promoters by remodeling the extracellular matrix and activating signaling pathways, others can function as tumor suppressors. Prostasin is a glycosylphosphatidylinositol-anchored serine protease that is expressed in epithelial tissues, including the ductal epithelium of the breast. We found that prostasin protein expression is lost in high-grade, poorly differentiated, invasive ductal carcinoma in both mice and humans. To test whether prostasin impacts tumor progression and metastasis, prostasin-deficient mice were crossed into the oncogene-induced transgenic MMTV-PymT mammary tumor model. While prostasin deficiency did not affect primary tumor growth, it resulted in a significantly increased spontaneous dissemination of cancer cells to the lungs, suggesting a causal relationship between the loss of prostasin expression and progression to distant metastasis of breast cancer. At the cellular level, re-expression of prostasin in human breast cancer cells that have lost endogenous prostasin attenuated their invasive properties. Importantly, silencing prostasin expression in non-transformed human mammary epithelial cells (HMECs) resulted in the disruption of epithelial integrity and the loss of tight junctions (TJs), an early hallmark of cells acquiring an invasive phenotype. Discovery proteomics identified HMEC-expressed fibronectin (FN) as a regulatory target of prostasin and revealed increased levels of FN upon prostasin silencing. Mechanistically, cellular FN plays a causal role in TJ integrity in HMECs, and concomitant silencing of FN and prostasin rescues the defects caused by prostasin loss. Prostasin-mediated FN regulation represents a novel mechanism for regulating mammary epithelial cell TJ integrity and a potential candidate pathway for targeted therapy in breast cancer patients.

## Introduction

Extracellular proteases are frequently upregulated in breast cancer and have long been associated with the promotion of metastatic disease and poor prognosis [1–7]. Interestingly, studies in recent years have revealed that some proteases exhibit tumor-suppressive effects and that their expression is lost during carcinogenesis, thus challenging the paradigm that proteases act solely as culprits in cancer. Prostasin, a glycosylphosphatidylinositol (GPI)-anchored serine protease, plays complex and context-dependent roles in cancer [1, 8, 9]. That is, prostasin acts as a tumor suppressor and is frequently lost during progression in some cancer types, while it displays pro-oncogenic properties and is overexpressed in other cancer types. Prostasin (encoded by the *PRSS8* gene) was first identified in the seminal fluid and the prostate gland and is widely expressed in epithelial cells across multiple organs, including the kidneys, lungs, colon, and epidermis [1, 8–10]. Functionally, prostasin is involved in a range of processes, including sodium and fluid balance, blood pressure control, airway hydration, and epidermal barrier function [1, 8, 9]. The role of prostasin in breast cancer progression and metastasis in vivo has not been explored, and we therefore conducted a comprehensive study utilizing a genetic mouse mammary carcinoma model in combination with a prostasin deficiency model. We demonstrate that prostasin acts as a suppressor of distant metastasis without a significant effect on primary mammary tumor size. At the cellular level, prostasin impairs invasion in breast cancer cells and is critical for maintaining integrity in non-cancerous mammary epithelial cells via tight junctions (TJs). Tight junctions are protein complexes that maintain the structure and function of epithelial tissues by controlling cell-cell adhesion, barrier formation, epithelial permeability, and cell polarity [11]. Disrupted cell-cell connections due to tight junction loss may contribute to cancer cell detachment and invasion, a crucial step in the metastatic cascade [12]. Unbiased proteomic analysis in human mammary epithelial cells (HMECs) revealed that loss of prostasin led to increased levels of the cellular fibronectin (FN) protein, a high-molecular weight glycoprotein with essential roles in cell adhesion, migration, and differentiation in physiological processes such as embryonic development and wound healing [13]. Aberrant regulation of fibronectin is associated with several diseases, including cancer [14, 15]. Our functional investigations revealed that increased FN levels were directly implicated in disrupting the TJ integrity in HMECs. We report tumor and metastasis suppressive roles for prostasin in cellular and mouse models of breast cancer, and the identification of a novel molecular mechanism by which prostasin regulates cellular FN to maintain tight junction integrity of mammary epithelial cells.

## Results

### Prostasin protein is lost in high-grade breast cancer

**Fig. 1:**
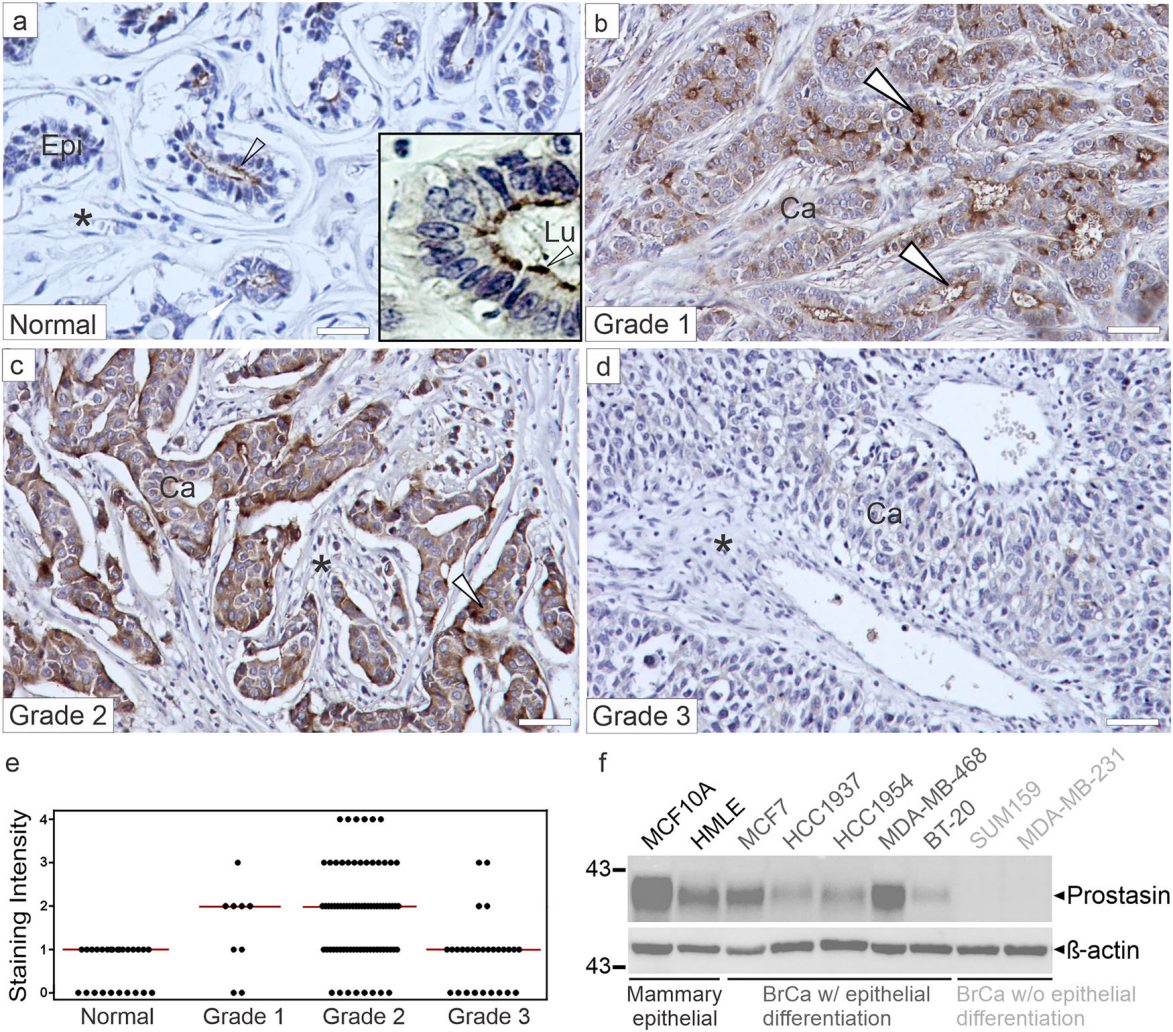
Prostasin protein expression is lost in high-grade invasive ductal carcinoma. Representative prostasin immunohistochemistry in human breast tissues. **a** Cell-surface expression in luminal epithelium cells of normal breast tissue (arrowheads) with apical membrane staining of the polarized cells (arrowhead in high magnification insert). Lu = lumen of mammary duct. **b** In grade 1 IDC, cells have partially lost polarization, but prostasin is still primarily detected on the cell surface of carcinoma cells. **c** In grade 2 lesions, prostasin displays a mostly diffuse staining with minimal cell-surface staining, **d** whereas prostasin protein is altogether undetectable or present at very low levels in poorly differentiated grade 3 lesions. Size bars = 50 μm. Epi = normal epithelium, Ca = carcinoma cells, asterisk = stromal tissue. **e** Intensity of staining was assessed manually using defined scoring criteria (0–4). Each dot represents samples from individual patients (*N* = 26 Normal/cancer adjacent normal, *N* = 9 grade 1, *N* = 76 grade 2, and *N* = 28 grade 3). Significant differences are observed between normal versus grade 2 (*P* < 9.0 × 10^−7^) and grade 2 versus grade 3 (*P* < 1.9 × 10^−^^4^). Kruskal-Wallis ANOVA with Dunn’s post hoc test was used to determine statistical significance. Grade 3 is not significantly different from normal. Red horizontal line = median. **f** Western blot analysis of prostasin expression in whole-cell lysates from non-transformed mammary epithelial cells (black font), breast cancer cell lines that still express epithelial markers including E-cadherin (dark grey font), and breast cancer cell lines that have lost epithelial markers (light gray font).

As part of our focus on membrane-anchored serine proteases (MASPs) and their role in breast cancer progression, we previously identified two members of the Type II transmembrane serine protease (TTSP) family, matriptase and TMPRSS13, as tumor promoters in murine genetic models of invasive ductal carcinoma (IDC) [16, 17]. While prostasin has been reported to be expressed in breast cancer cell lines and tumors (reviewed in [9]), no systematic studies of its protein expression during progression have been published. We therefore performed expression analysis of prostasin localization and levels using tissue arrays containing human IDC samples and normal breast tissues using immunohistochemistry (IHC) (Fig. 1). A monoclonal anti-prostasin antibody with previously validated specificity was used [18, 19]. In normal human breast tissues, the prostasin staining intensity was uniformly confined to the epithelial cells, primarily at the apical cell membrane, with no detectable staining in the stroma (Fig. 1a). In IDC, prostasin was widely expressed in the cancer cells, mainly localized to the cell surface of well differentiated (grade 1) tumors (Fig. 1b). In moderately differentiated (grade 2) cancers, prostasin expression was generally more diffuse with less defined cell-surface staining and more pronounced intracellular localization (Fig. 1c). When primary antibodies were substituted with non-immune rabbit IgG in serial sections of all the samples, no significant staining was observed (Supplementary Figure. S1). Importantly, prostasin expression was low or undetectable in poorly differentiated IDC (grade 3), demonstrating a loss of prostasin expression in tumors of higher grades (Fig. 1d). The staining intensity was scored on a scale from 0 to 3 (see “Materials and Methods”), and statistical analyses showed a significant decrease in staining intensity in high-grade IDC compared to normal tissue and low-grade IDC (Fig. 1e). In cultured human non-transformed mammary epithelial cells (MCF10A, HMLE [20, 21]), prostasin protein was readily detected by western blot analysis (Fig. 1f, two left lanes). Breast cancer cell lines with epithelial differentiation, *e.g*., expressing E-cadherin (MCF7, HCC1937, HCC1954, MDA-MB-468, BT-20), also expressed prostasin, whereas cell lines that have lost epithelial differentiation and acquired mesenchymal features have lost detectable prostasin (SUM159, MDA-MB-213, two right lanes) [18, 22]. Together, these observations indicate a correlation between the presence of prostasin and the differentiated state of cells in the mammary gland epithelium.

### Loss of prostasin expression during progression in the MMTV-PymT model of breast cancer

**Fig. 2:**
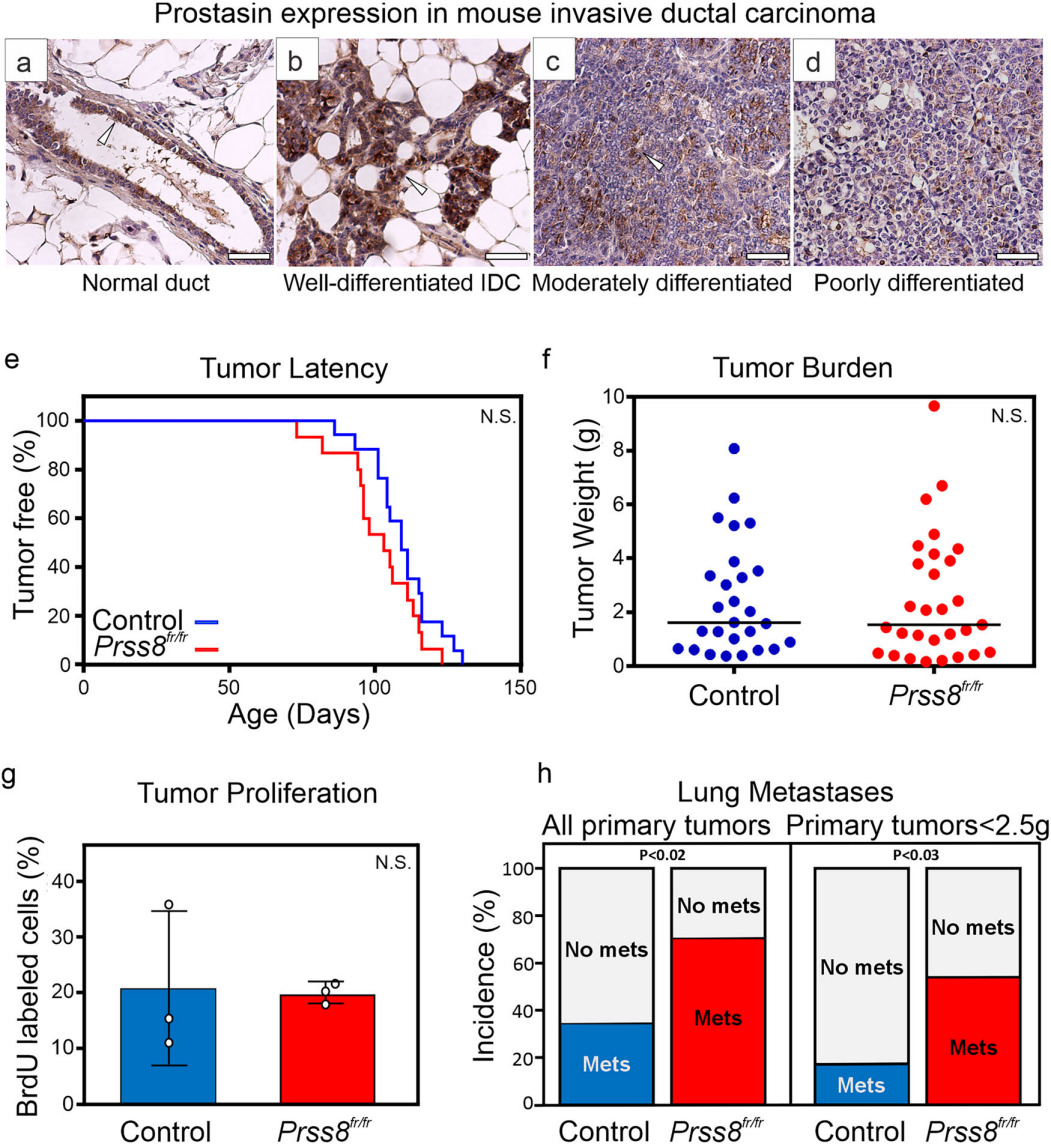
Significant increase in lung metastasis in *PRSS8*^*fr/fr*^ mice without change in tumor latency, burden, and proliferation. **a**–**d** Endogenous prostasin visualized by IHC in *PRSS8*^+/+^/MMTV-PymT mammary glands. **a** Moderate staining in all ductal epithelial cells in the normal gland. **b** Strong staining in well-differentiated mammary lesions with gradual loss in moderately **c** and **d** poorly differentiated tumors. Size bars = 100 μm. **e** The Kaplan-Meier tumor-free curves of a prospective cohort of littermate female control mice (*N* = 17, blue line) and *PRSS8*^*fr/fr*^ mice (*N* = 19, red line) of the first palpable mammary mass (tumor latency). The Mantel-Cox log-rank test was performed to assess statistical differences in tumor latency. No significant difference (N.S.). **f** Mammary tumor weight at 132 days in individual littermate control (*N* = 27, blue dots) and *PRSS8*^*fr/fr*^ mice (*N* = 29, red dots). Horizontal lines = median. Mann-U two-tailed statistical test performed for tumor burden analysis. No significant difference (N.S.). **g** Carcinoma cell proliferation in 132-day-old mice. Quantification of the number of BrdU-positive, proliferating carcinoma cells relative to total carcinoma cell number in mammary tumors. Positive and negative cells were counted in 20x microscopy fields by an investigator who was unaware of genotypes. Mean relative BrdU-positive cells in tumors from control mice (*N* = 3, blue bar), and littermate *PRSS8*^*fr/fr*^ mice (*N* = 3, red bar). Error bars represent S.D. An unpaired two-tailed t-test was used for tumor proliferation analysis. No significant difference (N.S.). **h** Lungs from tumor-bearing control mice and *PRSS8*^*fr/fr*^ mice were resected at 132 days. The presence of lung metastases was determined by inspection using a dissection microscope and histologically by evaluating multiple H&E-stained sections from paraffin-embedded lungs. For all tumor-bearing mice, 10/27 (37%) of control mice and 20/29 (69%) of *PRSS8*^*fr/fr*^ mice had lung lesions (*P* < 0.02). For mice with a total tumor burden of 2.5 grams (g) or below, 3/17 (18%) of control and 10/19 (53%) of *PRSS8*^*fr/fr*^ mice had lung lesions (*P* < 0.03). Chi-square test was used to assess differences in lung metastasis.

The transgenic mouse mammary tumor virus (MMTV) Polyoma middle T (PymT) mice develop multifocal mammary carcinomas with tumor progression that is very similar to that seen in human IDC [23, 24]. To determine if prostasin expression profiles in the MMTV-PymT cancer model closely mimic those observed in human breast cancer, the expression of prostasin in the normal mouse mammary glands and in the mouse mammary tumors was characterized by IHC (Fig. 2a–d). Similar to the phenotypes of humans, prostasin was expressed in the epithelial cells of the normal mammary gland (Fig. 2a), in the well-differentiated tumors (Fig. 2b), and the moderately differentiated tumors (Fig. 2c), with a loss of expression in the poorly differentiated tumors (Fig. 2d). These prostasin expression patterns align well with those observed in the human patient samples as described above and validate the MMTV-PymT mouse model as a relevant preclinical model for human breast cancer suitable for investigating the consequences of prostasin loss-of-function.

### Prostasin deficiency increases lung metastasis incidence in the MMTV-PymT model of breast cancer

As described above, prostasin is lost during mammary tumor progression/dedifferentiation in both humans and mice. To assess whether there is causality in the observed relationship between prostasin expression and tumor differentiation, the effects of prostasin deficiency during breast cancer progression in vivo were interrogated. Prostasin-null mice succumb shortly after birth due to severe defects in the cornified layer of the epidermis, which leads to the loss of a water-retaining barrier and fatal dehydration [25]. To circumvent this, we utilized a genetic mouse model (*Prss8* ^*fr/fr*^) [26] harboring a missense point mutation (*frizzy* variant, *fr*) in the murine prostasin gene, *Prss8*. A single point mutation in exon 4 of *Prss8* leads to an approximate 95% reduction in the catalytic activity of the murine prostasin and the inability of the mutated protease to form the enzymatic activity-dependent covalent complex with the serpin, protease nexin-1 (PN-1) [26, 27]. The *Prss8*^*fr/fr*^ mice present with sparse pelage hair and curly vibrissae with no reported differences in growth, life span, or fertility [26, 27]. We did not detect any overt defects in the *Prss8*^*fr/fr*^ females regarding mammary gland development based on whole mount analysis and lactation competency (Supplementary Fig. S2). Thus, these mice are well-suited to study the progression of breast cancer in mammary glands with a significantly reduced prostasin activity. For this purpose, prospective cohorts with the *PymT*-*Prss8*^*fr/fr*^ and the littermate control (*PymT-Prss8*^*+/+*^ *or PymT-Prss8*^*fr/+*^, together referred to as *PymT-Prss8*^*+*^) virgin female mice were established and examined weekly to determine the appearance of the first palpable mammary mass (primary tumor latency) (Fig. 2e). At 132 days of age, the total postmortem tumor burden was calculated as the total weight of all excised mammary glands in individual mice (Fig. 2f). No significant difference in tumor latency or total burden was detected between *Prss8*^*fr/fr*^ mice and littermate control mice. The percentage of proliferating carcinoma cells was determined by IHC detection of bromodeoxyuridine (BrdU) upon intraperitoneal injection of the synthetic thymidine analog 2 hours prior to euthanasia (Fig. 2g). No significant difference in proliferation was detected, indicating that prostasin deficiency does not contribute significantly to the growth of primary mammary tumors. The presence of lung metastases was determined by macroscopic and microscopic examination of lung surfaces and histological examination of lung sections. Interestingly, a significant increase in lung metastasis incidence was observed in the *PymT-Prss8*^*fr/fr*^ (20/29, 69%) in comparison to the *PymT-Prss8*^*+*^ mice (10/27, 37%) (Fig. 2h, left panel). An association between primary tumor size and metastasis incidence is observed in mouse models and in many human cancers, including breast cancer [28, 29]. Therefore, we also compared mice with a tumor burden of 2.5 grams or below to determine the significance of low prostasin for metastasis in mice with smaller primary tumors. The overall metastasis incidence was lower in mice with a lower primary tumor burden; however, the incidence in the *PymT-Prss8*^*fr/fr*^ was close to three times higher (10/19, 53%) than in the *PymT-Prss8*^*+*^ control mice (3/17, 18%) (Fig. 2h, right panel). These in vivo studies suggest that prostasin suppresses the progression of breast tumors into metastatic disease in a preclinical model.

### Prostasin re-expression impairs breast cancer cell invasive potential

**Fig. 3:**
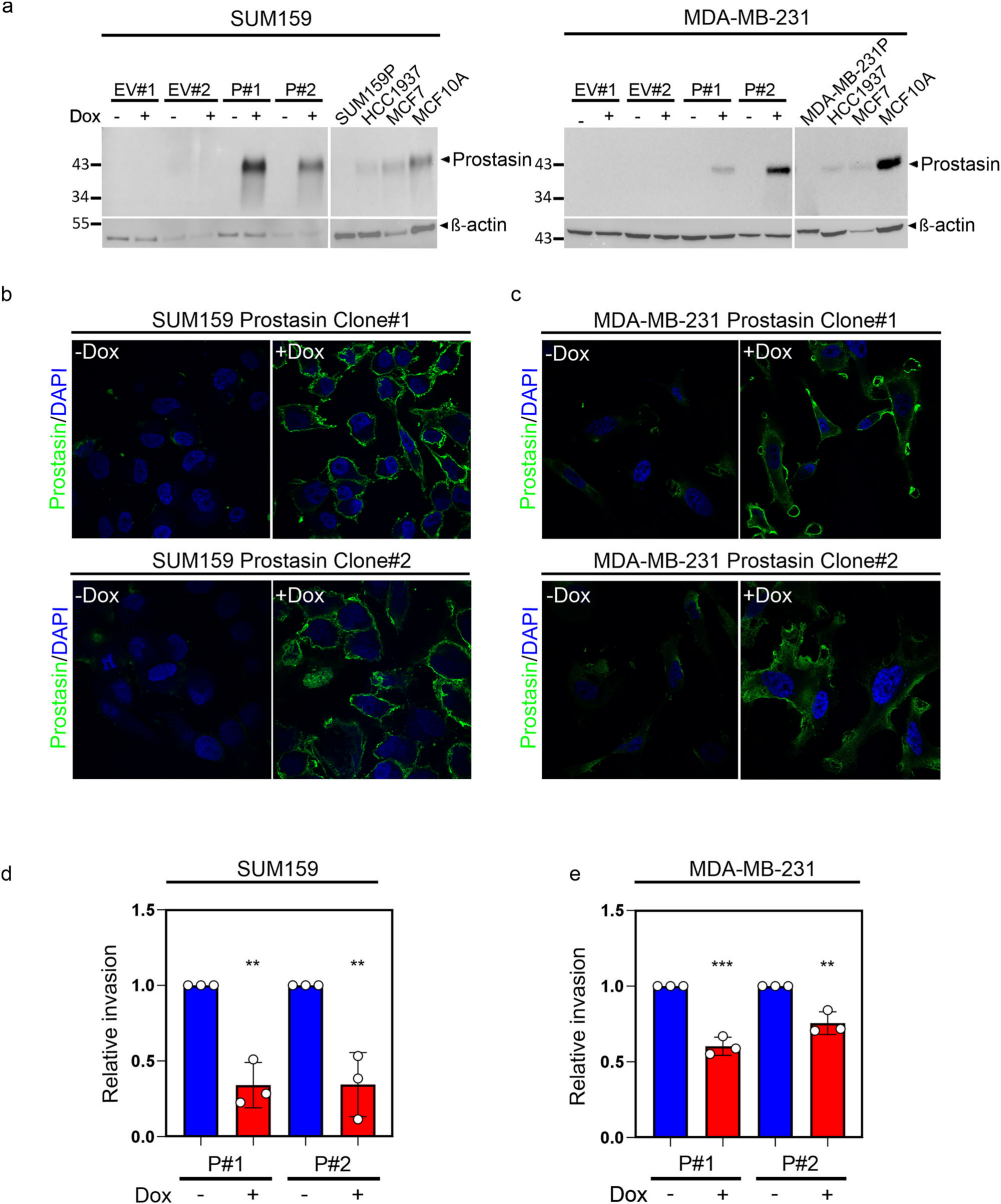
Re-expression of prostasin to the cell surface in breast cancer cell lines impairs invasion. **a** Western blot analysis of SUM159 cells (left) and MDA-MB-231 cells (right) with inducible prostasin expression of the pLenti4/TO/V5-DEST harboring either no insert (empty vector; EV) or human prostasin (P) cDNA after 48 h with or without 100 ng/mL doxycycline (Dox) treatment. Two stable independent clones are shown for each cell line, with the endogenous prostasin-expressing controls HCC1937, MCF10A cells, and prostasin-deficient SUM159P (P = Parental) or MDA-MB-231P cells. ß-actin used as loading control. Two independent clones for each of the SUM159 **b** and MDA-MB-231 cell lines **c** with inducible stable transfection of prostasin WT were treated for 72 hours with Dox (+Dox) or without doxycycline (−Dox) before seeding onto glass coverslips. 48 hours after, cells were fixed (no permeabilization), incubated overnight with rabbit-anti prostasin antibody, and analyzed by confocal microscopy (LSM 780 Zeiss). Merged images of nuclei (DAPI, *blue*) and prostasin (*green*) are shown. Reconstituted basement-membrane invasion assay. Two stable independent clones for each of the SUM159 **d** and MDA-MB-231 cell lines **e** transfected with inducible prostasin with (+, red bars) or without Dox (−, blue bars) for 72 h prior to invasion for 16 h are shown. Data from 3 independent experiments in triplicate for each clone, normalized to control conditions, are shown. (**P < 0.01, ***P < 0.001). Error bars represent S.D. An unpaired two-tailed t-test was used to determine statistical significance.

The capability of cancer cells to disseminate and invade surrounding tissue is critical for the formation of metastases. Our genetic breast cancer mouse model revealed an increase in lung metastases in mice with prostasin deficiency compared to control mice. To assess if restoring prostasin in aggressive human breast cancer cell lines that have lost endogenous prostasin would affect their ability to invade, we performed extracellular matrix invasion assays. Plasmid-mediated expression of human prostasin cDNA was performed in the SUM159 and the MDA-MB-231 human breast cancer cell lines, presenting a mesenchymal-like phenotype with high motility/invasion capability. The empty vector (EV) without a cDNA insert was used as a control. Neither of these cell lines express endogenous prostasin nor the epithelial marker E-cadherin (Fig.1f and [18, 22]). A doxycycline (Dox)-inducible system was used to circumvent artifacts due to selection bias from prostasin expression, potentially affecting cellular survival. Multiple independent clones were used to minimize potential clone-specific artifacts. As expected, no prostasin expression was detected by western blot analysis upon transfection with the EV (Fig. 3a, both panels, left lanes). The SUM159 cells transfected with prostasin displayed a robust prostasin expression upon Dox treatment with no detectable prostasin in vehicle-treated cells (Fig. 3a, left panel). The MDA-MB-231 cells also displayed prostasin expression under Dox treatment only (Fig. 3a, right panel). Cell-surface localization of Dox-induced prostasin was confirmed by immunocytochemistry (ICC) (Fig. 3b, c) with no detection of prostasin upon vehicle treatment (Fig. 3b, c, left panels), in agreement with the western blot data. Re-expression of prostasin in both the SUM159 cells (Fig. 3d) and the MDA-MB-231 cells (Fig. 3e) significantly impaired their ability to traverse the reconstituted basement membrane upon Dox-induction. No effect on invasion upon Dox treatment was detected in either cell line transfected with the EV instead of prostasin (supplementary Fig. S3). These observations suggest that restoring cell-surface prostasin in breast cancer cells that have lost epithelial differentiation suppresses their highly invasive phenotype.

### Loss of prostasin in non-transformed mammary epithelial cells causes loss of tight junctions

**Fig. 4:**
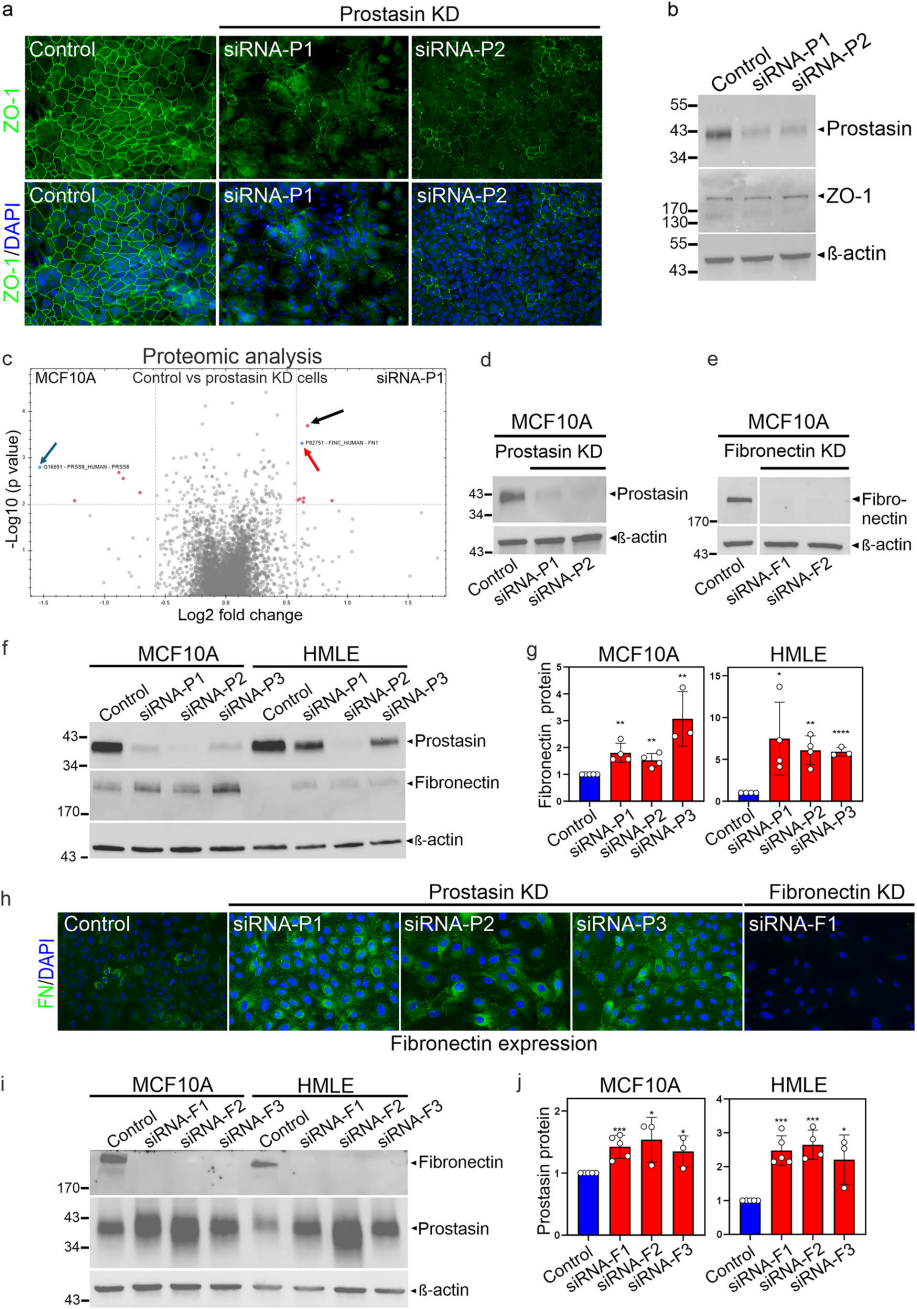
Prostasin silencing in human mammary epithelial cells disrupts tight junction integrity and causes increased levels of cellular fibronectin. **a** MCF10A cells were transfected with control siRNA (scrambled %GC matched) or two prostasin siRNAs (siRNA-P1 or P2) for 72 hours at the time of seeding. Cells were fixed, permeabilized, incubated overnight with anti-ZO1 antibody, and analyzed by fluorescent microscopy. Nuclei (DAPI, *blue*), ZO-1 (*green*). Diffuse or punctate ZO-1 staining indicative of disrupted tight junctions is observed upon prostasin silencing. Representative of five independent experiments. A different experiment using three prostasin siRNAs is shown in Fig. S4. **b** Corresponding western blot analysis of prostasin, ZO-1, and β-actin upon silencing with two non-overlapping siRNA duplexes targeting prostasin (siRNA-P1 or P2) compared to control siRNA (scrambled %GC matched). **c** MS-based quantitative proteomic analysis comparing the proteomes of MCF10A cells transfected with non-targeting scrambled control siRNA and siRNA-mediated knockdown of prostasin. Data for siRNA-P1 is shown and data for siRNA-P2 is shown in Supplemental Fig. S5. Blue arrow= *PRSS8/*Prostasin (*P*  = 0.0016), red arrow = *FN*/Fibronectin (*P* = 0.0005), black arrow = *MAD1L/* Mitotic Arrest Deficient 1 Like 1 (*P* = 0.0002) **d** Western blot analysis of lysates used for proteomic analysis of prostasin and β-actin (loading control) upon silencing with two different siRNA duplexes (siRNA-P1 and siRNA-P2) targeting prostasin in MCF10A cells. **e** Specific detection of cellular fibronectin was validated using a rabbit anti-fibronectin antibody in control cells and cells transfected with non-overlapping siRNAs that target fibronectin (siRNA-F1 and siRNA-F2). **f** To validate fibronectin as a significantly differentially abundant protein in two different mammary epithelial cell lines, western blot analysis of MCF10A (left four lanes) and HMLE (right four lanes) was performed using cell lysates from cells transfected with three non-overlapping siRNAs targeting prostasin (siRNA-P1, siRNA-P2, and siRNA-P3). Blots were probed for fibronectin (middle panel) and β-actin (bottom panel, loading control). **g** Densitometric quantification of fibronectin levels in MCF10A (left graph) and HMLE (right graph) cell lines. Blue bars = control, red bars = prostasin knockdown. Data from 3 independent experiments for each cell line were normalized to β-actin. Asterisks indicate significant difference from scrambled %GC-matched control, *P < 0.05. **P < 0.01, ****P < 0.0001. An unpaired two-tailed t-test is used to determine statistical significance. Error bars represent S.D. **h** MCF10A cells were silenced with three non-overlapping siRNAs targeting prostasin (siRNA-P1, siRNA-P2, and siRNA-P3) or fibronectin (siRNA-F1 and siRNA-F2, latter not shown) for 72 hours. Cells were fixed and permeabilized, incubated overnight with rabbit anti-fibronectin antibody, and analyzed by fluorescence microscopy. Merged images of nuclei (DAPI, *blue*) and fibronectin (*green*) are shown. **i** Western blot analysis of fibronectin (top panel), prostasin (middle panel), and β-actin (bottom panel, loading control) upon silencing fibronectin with three non-overlapping siRNAs (siRNA-F1, siRNA-F2, and siRNA-F3). **j** Densitometric quantification of prostasin levels in MCF10A (left graph) and HMLE (right graph) cell lines. Blue bars=control, red bars=fibronectin knockdown. Data from at least 3 independent experiments for each cell line were normalized to β-actin. Asterisks indicate significant difference from scrambled %GC-matched control, *P < 0.05. **P < 0.01, ***P < 0.001. An unpaired two-tailed t-test was used to determine statistical significance. Error bars represent S.D.

Based on the findings that prostasin is expressed in normal mammary epithelial cells and lost in poorly differentiated breast cancer cells, along with the observation that the loss of prostasin in mice led to an increased metastatic spread of mammary cancer in mice, we set out to uncover tumor/metastasis-suppressing cellular properties regulated by prostasin. TJs are critical for normal epithelial cell function, and the loss of TJs is a hallmark of decreased epithelial integrity and gain of invasive properties [30]. To query whether prostasin plays a role in maintaining mammary epithelial differentiation and integrity, prostasin silencing was performed in the non-transformed mammary epithelial MCF10A cells, followed by visualization of the TJ protein zonula occludens-1 (ZO-1) by ICC (Fig. 4a and Supplementary Fig. S4). The control cells (left panels) displayed a highly organized and differentiated epithelial cell network where TJs appear as a crisp, polygonal “honeycomb” meshwork outlining the cell perimeters. In the prostasin-silenced cells, this network was less defined with a disrupted and punctate staining (middle and right panels), suggesting that prostasin is important for TJ assembly and/or maintenance. No differences in the total ZO-1 protein levels were detected by western blotting (Fig. 4b), suggesting that prostasin may regulate ZO-1 trafficking and localization rather than the expression levels. Interestingly, translocation of ZO-1 from cell junctions to the intracellular space has been linked to tumor progression and increased cell motility [31, 32]. These data imply that prostasin is essential for preserving TJs and epithelial integrity in mammary epithelial cells and that its absence may be an early causal factor in carcinogenesis.

### Discovery mass spectrometry reveals an increase in the cellular fibronectin protein upon prostasin silencing in mammary epithelial cells

To uncover molecular mechanisms underlying the requirement for prostasin to support TJ integrity in mammary epithelial cells, we analyzed changes in the proteomic landscape upon prostasin silencing in MCF10A cells. Discovery mass spectrometry data were visualized in a volcano plot to show the statistical significance and the magnitude of changes between the control and the two non-overlapping siRNAs: siRNA-P1 (Fig. 4c) and siRNA-P2 (Supplementary Fig. S5). As expected, the prostasin protein levels were significantly lower upon gene silencing (blue arrow in Fig. 4c and Supplementary Fig. S5), confirmed by using the same lysates in western blot analysis (Fig. 4d). Since multiple proteins were differentially expressed, our criteria for further study included 1) a significant change with both prostasin siRNAs, 2) the validation of the protein level difference by western blotting in MCF10A, and 3) an observation of similar changes in a second mammary epithelial cell line (HMLE). Two candidates fulfilled the first and second criteria: fibronectin (FN) (red arrow in Fig. 4c and Supplementary Fig. S5) and Mitotic Arrest Deficient 1-Like (MD1L1 or MAD1L1) (black arrow in Fig. 4c and Supplementary Fig. S5). High MAD1L1 protein expression was reported to be associated with poor prognosis in breast cancer and represents a potential candidate for future studies [33]. For this study, we chose to focus on FN because it displayed the most consistent increase in protein levels by western blot and ICC upon prostasin silencing in our cell culture models. The specificity of the anti-FN antibodies was confirmed using two non-overlapping siRNAs targeting FN (Fig. 4e and Fig. 4h, right panel). In multiple independent experiments using three prostasin siRNAs to silence prostasin, an increase in the FN protein level was observed in both the MCF10A and the HMLE cells by western blot analysis (Fig. 4f). Quantitative analysis revealed a significant increase of the FN protein in the MCF10A (2 to 3-fold) and the HMLE (6 to 7-fold) cells (Fig. 4g). Cellular FN is expressed by various benign and malignant epithelial and mesenchymal cells. Its complex role in cancer has mostly focused on the extracellular FN deposited by these cells as filaments into an extracellular matrix (ECM), and high levels of FN often correlate with poor prognosis [15]. In IDC, both the FN transcript and protein can be detected in stromal fibroblasts as well as carcinoma cells [34]. Interestingly, both stromal and carcinoma cell FN expression is correlated with tumor aggressiveness (lymph node stage, histologic grade, lymphovascular invasion, and HER2 positivity), but only carcinoma cell FN expression is an independent prognostic factor of disease-free survival [35]. In cultured cells, previously published studies have demonstrated that mammary epithelial cells, including MCF10A, display low levels of cellular FN by ICC compared to breast cancer cell lines such as MBA-MD-231 cells [36]. In control MCF10A cells, we similarly observed sparse and low-intensity FN staining (Fig. 4h, left panel). Upon prostasin silencing, an increased FN staining intensity was evident, with cells displaying both punctate and fibril-like staining (Fig. 4h, middle panels). No staining was detected in control cells transfected with siRNA targeting FN (Fig. 4h, right panel). To further evaluate the potential regulatory interplay between prostasin and FN, we silenced FN expression and measured the accompanying prostasin protein levels. We observed a significant increase in prostasin protein upon FN knockdown in both cell lines (Fig. 4i, j), suggesting that prostasin and FN participate in a bidirectional, mutual negative feedback loop. To investigate whether the prostasin/FN expression regulation occurred at the transcription level, RT-qPCR analysis was performed. Interestingly, we observed no significant change in FN mRNA levels in MCF10A or HMLE cells upon prostasin silencing (Supplementary Fig. S6a). Indeed, it has been reported that FN expression in cancer is regulated by various post-transcriptional mechanisms, including alternative splicing and a range of post-translational modifications (PTMs), including proteolytic processing (see Discussion). In contrast, significantly increased prostasin mRNA levels were detected in both cell lines upon FN silencing, thereby pointing to a transcriptional control within this arm of the regulatory feedback loop (Supplementary Fig. S6b).

### Mutually exclusive FN and ZO-1 expression in mammary epithelial cells

**Fig. 5:**
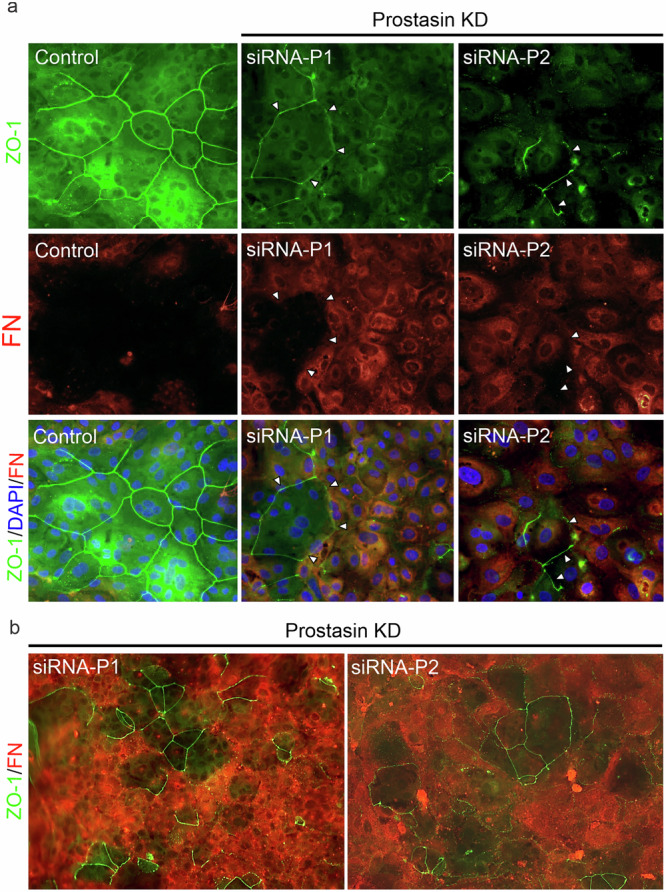
Mutually exclusive expression of ZO-1 and fibronectin in human mammary epithelial cells. **a** MCF10A cells were transiently transfected with scrambled %GC-matched control siRNA (left panels) or siRNAs silencing prostasin (P1 and P2, right panels) for 72 hours at the time of seeding. Cells were fixed, permeabilized, incubated overnight with anti-ZO-1 (upper panels) and anti-fibronectin antibodies (middle panels), and analyzed by fluorescent microscopy. Nuclei (DAPI, *blue*), ZO-1 (*green*), fibronectin (*red*). Merged ZO-1, FN, and DAPI, lower panels. Cells with strong cellular FN signal generally displayed low ZO-1 staining and in cells with minimal FN staining, cell-surface expression of ZO-1 was detected (illustrated with white arrowheads). Representative of five independent experiments. **b** ZO-1 and FN staining in prostasin-silenced MCF10A with artificial color enhancement to illustrate the mutually exclusive patterns of expression. The original photos with scrambled siRNA and FN silencing controls are included in Fig. S7.

To further explore the regulatory and functional FN-prostasin axis and its potential relevance for TJ integrity, co-staining of FN and ZO-1 by ICC was performed with visual analysis to compare their localization patterns between control cells and prostasin-silenced MCF10A cells (Fig. 5 and Supplementary Fig. S7). As expected, control cells displayed low FN expression with a prominent, organized ZO-1 staining (Fig. 5a and Supplementary Fig. S7a, left panels). Upon transient siRNA-mediated prostasin silencing by transfection, the majority of cells displayed a visibly increased FN staining, accompanied by a loss of ZO-1 staining. Remarkably, a subset of cells displayed low-level FN expression (cells that escaped prostasin silencing), and these cells had distinct ZO-1 TJ localization (Fig. 5a, b and Supplementary Fig. S7a, right panels). This suggests that FN and ZO-1 mutually repress the expression of each other and may represent opposing roles in the preservation of intact epithelial structure and function.

### Simultaneous silencing of FN and prostasin rescues the tight junction phenotype in mammary epithelial cells

**Fig. 6:**
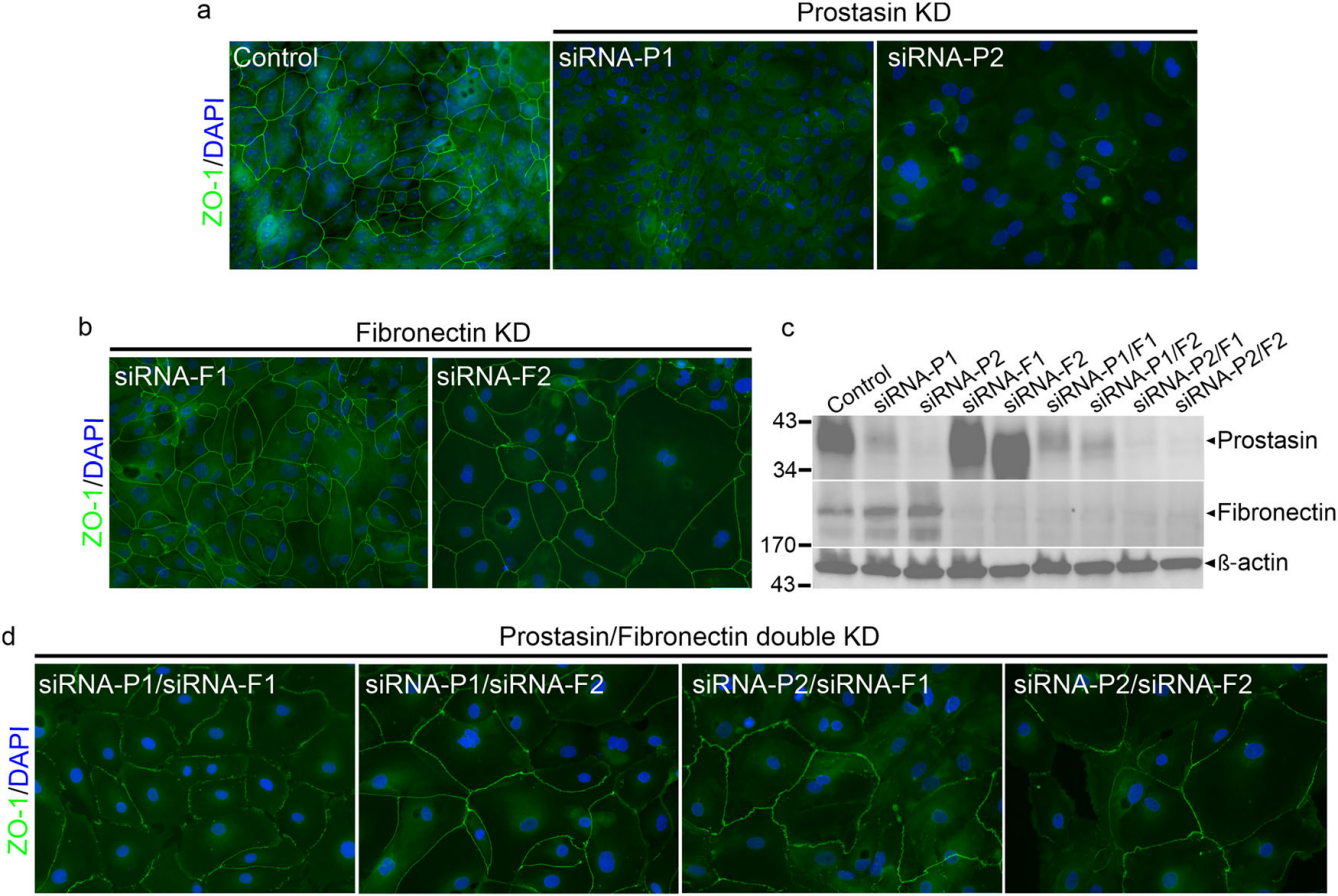
Concomitant silencing of FN and prostasin rescues the TJ defects caused by prostasin loss. **a** MCF10A cells were transfected with control siRNA (scrambled %GC matched) (left) or two non-overlapping prostasin siRNAs (siRNA-P1 and P2) (right), **b** two non-overlapping FN siRNAs (siRNA-F1, siRNA-F2), or **d** four different combinations of prostasin/FN siRNA. Cells were fixed, permeabilized, incubated overnight with anti-ZO-1 antibody, and analyzed by fluorescent microscopy. Nuclei (DAPI, *blue*), ZO-1 (*green*). **c** Corresponding western blot detection of prostasin, FN, and β-actin. Disrupted ZO-1 cell surface staining is observed upon prostasin silencing **a**, whereas clear continuous ZO-1 staining is present upon FN silencing and prostasin/FN silencing **b**, **d**. Representative of five independent experiments. A different independent experiment is shown in Fig. S8.

A regulatory relationship between prostasin and FN was indicated based on the data above. To query whether a functional relationship related to TJ integrity and epithelial cell function between the two exists, we compared the phenotypes between MCF10A cells where prostasin and FN were silenced either individually or simultaneously (Fig. 6 and Supplementary Fig. S8). Protein levels in the corresponding whole cell lysates were analyzed by western blotting to validate the expected decrease in the protein levels of the single and combination knockdown conditions (Fig. 6c). Single knockdown of either FN or prostasin again led to an increase of protein levels of the other, while cells that were transfected with each of the four combinations of siRNA for dual knockdown displayed low levels of both proteins. Silencing of prostasin caused the loss of defined ZO-1 staining as predicted (Fig. 6a and Supplementary Fig. S8a, right panels), whereas cells with FN silencing displayed well-defined ZO-1 staining comparably to that observed in control cells (compare left panels in Fig. 6a and Supplementary Fig. S8a with Fig. 6b and Supplementary Fig. S8b, respectively). Silencing both prostasin and FN markedly improved the assembly of ZO-1 into continuous tight junction structures along the cell borders (Fig. 6d and Supplementary Fig. S8c), a marked difference from the sparse, punctate staining seen with the prostasin single-gene silencing. The observed rescue effect from the dual disruption of prostasin and FN provides evidence that the two proteins have a significant, opposing functional role for TJ integrity. It should be mentioned that the ZO-1 cellular outlines in the double knockdown cells appeared marginally less crisp and, in some areas, had a slightly more irregular appearance than in control cells, suggesting an incomplete rescue effect. This could be attributed to an incomplete FN silencing (Fig. 6c) or, alternatively, that additional prostasin-mediated mechanisms exist independently of its regulation of FN expression. We show that the regulatory interplay between prostasin and FN is critical for preserving proper ZO-1 localization to maintain the integrity of mammary epithelial cells.

## Discussion

We report that prostasin expression is lost in human and murine breast cancer, and that prostasin deficiency enhances breast cancer progression in vivo, causing increased incidence of lung metastasis. At the cellular level, the forced re-expression of prostasin in aggressive human breast cancer cells that have lost endogenous prostasin impaired their capacity for invasion. In non-transformed human mammary epithelial cells, RNAi-mediated downregulation of prostasin disrupted the typical continuous, circumferential ZO-1 localization widely recognized as an indicator for TJ integrity. To gain mechanistic insight into the functions of prostasin in mammary epithelial cells, proteomic profiling via unbiased mass spectrometry was conducted to identify differentially regulated proteins. Fibronectin (FN) was identified and verified in two mammary epithelial cell lines as being elevated in expression upon prostasin silencing. Interestingly, FN and prostasin exhibited a reciprocal regulatory mechanism, where the silencing of one resulted in an increase at the protein level of the other.

FN is a high molecular weight glycoprotein that interacts with a variety of molecules that mediate multiple functions, including ECM assembly, cell attachment, and cell motility [15, 37]. Of particular interest for our study, analysis of patient samples has shown that breast carcinomas with distant metastasis frequently have cancer cells expressing intracellular FN and that FN expression correlates with poor prognosis [35, 38]. Experimentally, the role of FN expressed by breast cancer cells was directly assessed using shRNA silencing of FN in MDA-MB-231 cells in two experimental xenograft models [39]. Mice receiving FN knockdown breast cancer cells via tail vein injection had a decreased total tumor burden, which prolonged survival in comparison to mice receiving control cells [39]. Additionally, injection of cancer cells into the tibia (a model of bone metastatic lesions) showed decreased lesion sizes in mice receiving FN knockdown cells versus control cells [39]. In cell culture assays, overexpression of the extradomain-B FN, an alternatively spliced isoform of FN, was associated with increased invasion of breast cancer cells [40]. Collectively, this indicates that dysregulation of FN plays a causal role in breast cancer progression. We propose that one mechanism by which prostasin exerts its tumor/metastasis suppressive effects is by regulating FN protein levels. We hypothesize that under homeostatic (pre-cancerous) conditions, FN and prostasin maintain physiological levels via a balanced, intact feedback loop, which becomes disrupted in cancer. FN expression in cancer is regulated by complex transcriptional, post-transcriptional, and post-translational mechanisms [15]. In a study comparing gene and protein expression by combined cDNA microarray and tissue microarray IHC analysis in breast cancer patient samples, significant increases in FN protein levels were detected in lymph node metastases compared to primary tumors, without a significant corresponding increase in FN transcript levels [41]. Our RT-qPCR data suggested that prostasin silencing did not alter FN transcript levels, suggesting a post-translational regulation. We are currently investigating the mechanism by which prostasin regulates FN protein levels and have observed that a purified recombinant prostasin can cleave/degrade FN in a cell-free system (LM Chen and KX Chai, unpublished data). Other potential mechanisms to be explored include the ubiquitin-proteasome system and lysosomal degradation, processes previously shown to be involved in FN turnover in different cellular settings [42]. We observed that TJ integrity was compromised by prostasin silencing in mammary epithelial cells and that concomitant silencing of FN restored ZO-1 localization. This suggests that FN and prostasin are components of a shared pathway for TJ regulation in this cellular model. The precise mechanisms by which FN affects TJs are still under investigation. One potential mechanism is FN-stimulated epithelial-mesenchymal transition (EMT) since, in addition to serving as a molecular marker for EMT, FN has been shown to promote this process [43, 44]. Thus, in human breast cancer MCF-7 cells, an epithelial non-invasive cell line that expresses adherens and tight junction proteins, stimulation with exogenous FN leads to downregulation of E-cadherin and ZO-1, and the upregulation of mesenchymal N-cadherin and vimentin [44]. Additionally, FN promoted cell migration and invasion, with increased expression of calpain-2 and proteolysis of focal adhesion kinase 1 (FAK) [44]. While we did not observe any consistent markedly different protein levels of E-cadherin, vimentin, or FAK by western blotting or in our mass spectrometry analysis (JG Lundgren and K List, unpublished data) in MCF10A cells upon prostasin silencing, an effect on EMT cannot be ruled out. First, our results showing increased FN and a robust ZO-1 disruption phenotype after 72 hours of transient prostasin silencing may indicate an early or partial EMT, whereas the incomplete prostasin knockdown (80-90%) may not be sufficient to induce additional detectable changes associated with a mesenchymal phenotype in this time frame. Second, the cellular experiments presented in the present study were carried out on surfaces void of protein coating and with no exogenous FN present. A published study demonstrated that MCF10A mammary epithelial cells grown in the monolayer culture on tissue culture plastics produce low levels of FN that is secreted into the culture medium and not assembled into a matrix [43]. Interestingly, when cells were grown on plates coated with FN, up-regulation of FN mRNA and protein expression was induced with a subsequent EMT response. The FN-induced EMT in this model depended on Src kinase and ERK/MAP kinase signaling [43]. In a different study, it was reported that endogenous FN expressed by MCF10A cells could be detected both cellularly and as deposits on the culture plates [42]. We plan to further delineate prostasin-FN-mediated cellular changes on different coating surfaces, as well as in 3D culture models, because studies of mammary epithelial cells and breast cancer cells have found that 3D culture conditions affect FN expression, deposition, and function in these cells [43, 45]. From a translational perspective, targeting tumor/metastasis suppressors holds therapeutic potential for cancer or cancer therapeutics along with unique challenges [46]. Since most extracellular proteases studied to date, including MASPs, have tumor/metastasis-promoting properties, targeted drug development strategies have focused on inhibitors to impair their proteolytic function [1]. Given the metastasis-suppressive properties of prostasin in breast cancer, inhibition of prostasin is not appropriate. Instead, strategies to restore prostasin expression and function could be explored, including delivery of exogenous protein, mRNA, or DNA to re-establish prostasin expression. In a model of bladder inflammation, a condition in which prostasin levels are decreased compared to healthy bladder, local delivery of liposomes containing a prostasin-expressing plasmid attenuated cytokine-inducible nitric oxide synthase expression [47]. A shared mechanism underlying the loss of prostasin expression in several cancer types, including breast cancer, was identified as hypermethylation of the *PRSS8* promoter region [48]. Novel CRISPR-based approaches now allow for the targeted DNA demethylation of specific endogenous gene loci in cell culture models [49]. Targeting FN in cancer has received increased attention in the past decade, and strategies include disrupting its pro-tumorigenic roles in the tumor microenvironment (e.g., by blocking FN-integrin interactions with antibodies or peptides), using its overexpression for targeted delivery of therapies (e.g., delivering cytotoxins via FN-targeting ligands), and targeting FN with CAR-T cells [50–53]. Challenges include the complex, context-dependent roles of FN and its essential function in normal tissue repair, requiring specific targeting of FN variants that are differentially expressed in cancer. While the approaches of re-establishing prostasin tumor suppressor function or targeting fibronectin in tumors may show therapeutic potential, their use in patients is complicated by technical challenges and safety precautions. Most extracellular proteases have multiple targets, and it is predicted that many proteases, including prostasin, mediate pleiotropic effects during cancer progression. This study highlights the continued need for basic scientific investigation into proteases and their complex functions to uncover novel targets for future cancer therapies.

## Materials and Methods

### Animals

All procedures involving live animals were performed following institutional guidelines and standard operating procedures. For details on the generation of experimental mice, genotyping, and cohort establishment, please see “Supplemental Materials and Methods.”

### Immunohistochemistry

Tissue arrays (BR803, BR2083, BR2085, BR2086) were from US Biomax, Inc/TissueArray.com (Derwood, MD). Antibodies used were mouse anti-human prostasin (612173, BD Biosciences, San Jose, CA) or rabbit anti-human prostasin (HPA030436, Millipore Sigma, Burlington, MA). As negative controls, non-immune rabbit IgG (12-370, Millipore Sigma) or mouse IgG2a (E5Y6Q, Cell Signaling, Danvers, MA) were used. Bound antibodies were visualized using ImmPRESS HRP Horse Anti-Mouse IgG Polymer Kit, Peroxidase (MP-7402-15, Vector Laboratories, Newark, CA) or ImmPRESS HRP Goat Anti-Rabbit IgG Polymer Kit, Peroxidase (MP-7451-15, Vector Laboratories). 3,3’-Diaminobenzidine (DAB) was used as the substrate (Millipore Sigma), and the arrays were counterstained with *hematoxylin* QS (Vector Laboratories). All microscopic images were acquired on a Zeiss Scope A.1 using digital imaging. Assessment of staining intensities was performed by evaluation by an investigator unaware of the tumor grades. Scores were assigned an arbitrary score (0-4) based on the intensity and extent of epithelial cell/cancer cell staining. For more information, see “Supplemental Materials and Methods”. In mouse tissues, prostasin was detected with mouse anti-human prostasin (612173, BD Biosciences) using the M.O.M. (Mouse on Mouse) Elite® Immunodetection Kit, Peroxidase (PK-2200, Vector Laboratories) according to the manufacturer’s instructions. 3,3’- DAB was used as the substrate (Millipore Sigma), and slides were counterstained with *hematoxylin* QS (Vector Laboratories). Cell proliferation was visualized by intraperitoneal injection of 100 µg/g BrdU (Millipore Sigma, Burlington, MA) 2 h before euthanasia. BrdU incorporation was detected with a rat anti-BrdU antibody (Accurate Chemical and Scientific Corporation). All microscopic images were acquired on a Zeiss Scope A.1 using digital imaging. For more information, see “Supplemental Materials and Methods”.

### Cell culture

Malignant (BT-20, MCF-7, HCC1937, HCC1954, MDA-MB-231, MDA-MB-468, SUM159) and non-malignant (MCF10A, HMLE, and HEK293FT) cells were cultured as described in “Supplemental Materials and Methods.”

### Immunocytochemistry

Fixed cells were incubated with primary antibodies (rabbit anti-ZO-1 (D6L1E) Alexa Fluor® 488, Cell Signaling, Danvers, MA; rabbit anti-FN1 (E5H6X) Alexa Fluor® 488 Cell Signaling, Danvers, MA; sheep anti-fibronectin (AF1918) R & D Systems Minneapolis, MN; rabbit anti-prostasin (PA5-80945) ThermoFisher, Waltham, MA) overnight at 4 °C. Unconjugated sheep anti-fibronectin coverslips were incubated with donkey anti-sheep secondary AlexaFluor ® 555 (A-21436 ThermoFisher), and unconjugated rabbit anti-prostasin coverslips were incubated with goat anti-rabbit secondary AlexaFluor® 488 (A32731, ThermoFisher). Fluorescence images taken with an Olympus BX53 (20X) and confocal images were acquired on the Zeiss LSM 780 scope (63X) at the Microscopy Imaging and Cytometry Resources Core at Wayne State University School of Medicine. Acquired images were edited and merged using ImageJ software. For detailed protocol, see “Supplemental Materials and Methods.”

### Invasion assays

SUM159 and MDA-MB-231 WT and EV prostasin cells were first seeded in 6-well tissue culture plates in full serum media treated with (+) or without (−) 100 ng/mL doxycycline (Sigma-Aldrich, St. Louis, MO) for 48 hours. Following treatment, 30,000 cells were seeded in triplicate onto transwell inserts (8.0 μM pore size, Corning, Corning, NY) pre-treated with Cultrex Basement Membrane Extract (R & D Systems) at 1 mg/mL in serum-free media. Inserts were placed in 24-well plates with the bottom chamber containing serum-supplemented media as a chemoattractant, and cells were cultured on inserts for 16 hours, after which invading cells were fixed and stained using Kwik-Diff (Siemens, Deerfield, IL). Images of inserts were acquired using an EZ4D Stereo Zoom microscope with a digital camera (Leica Microsystems, Buffalo Grove, IL). Invaded cells were quantified using ImageJ software.

### Western Blot

Proteins were separated by SDS-PAGE under reducing conditions using 10% Mini-Protean gels or Criterion TGX midi gels (Bio-Rad, Hercules, CA) and transferred onto PVDF membranes. Primary antibodies used for western blotting include mouse anti-human prostasin (mAb) (612173, BD Biosciences, San Jose, CA), mouse anti-ß-Actin (NB600-501, Novus Biologicals, Centennial, CO), rabbit anti-fibronectin (E5H6X, Cell Signaling), and rabbit anti-ZO-1 (D6L1E, Cell Signaling). Detailed protocol is provided in “Supplemental Materials and Methods.”

### RNAi-mediated gene silencing

Transient knockdown of prostasin and/or fibronectin in MCF10A and HMLE cells was performed using Lipofectamine RNAiMAX according to the manufacturer’s instructions (Invitrogen, Life Technologies, Inc, Waltham, MA) with % GC-matched negative controls. Stealth siRNA duplexes targeting prostasin and fibronectin were obtained from Invitrogen Life Technologies (HSS108631 corresponding to prostasin siRNA-1, HSS108633 corresponding to prostasin siRNA-2, and HSS183514 corresponding to prostasin siRNA-3; HSS103780 corresponding to fibronectin siRNA-1, HSS103782 corresponding to fibronectin siRNA-2, and HSS177362 corresponding to fibronectin siRNA-3). Transfections were performed with 1.5 μL of 20 μM siRNA in a 6-well plate format in complete cell media in the absence of penicillin/streptomycin.

### Lentiviral transfection

The ViraPower^TM^ T-REx^TM^ Lentivirus Expression System (Invitrogen, Waltham, MA) was used for the generation of replication-incompetent lentivirus stably expressing a recombinant human prostasin under doxycycline regulation with previously published plasmids [54, 55]. Detailed protocol is provided in “Supplemental Materials and Methods.”

### Mass spectrometry

LC-MS/MS analysis was performed using a Thermo Scientific Vanquish-Neo chromatography system with an Acclaim PepMap 100 trap column (100 µm × 2 cm, C18, 5 µm, 100 Å, Thermo Fisher, Waltham, MA), and a Thermo Scientific Easy-Spray PepMap RSLC C18 75 µm x 25 cm column (Thermo Fisher). Mass spectrometry data were processed with Spectronaut 19.0 (Biognosys, Schlieren, Switzerland), using the directDIA strategy and the Pulsar search engine. Spectra were searched against the Human Uniprot FASTA database downloaded March 30, 2021. The search parameters included trypsin with up to two missed cleavages. Variable modifications are oxidation of M and of protein N-termini by Acetylation, Met Loss, or both Acetylation and Met Loss. Carbamidomethylation of cysteine was a fixed modification. Quantification was accomplished using the MS2 data with default Spectronaut 19 settings. For the entire data set, the false discovery rate (FDR) was calculated using a cut-off of 1% for the identification of precursors, peptides, and protein groups. Detailed protocol is provided in “Supplemental Materials and Methods.”

### TaqMan RT-PCR analyses

MCF10A cells were reverse-transfected with % GC-matched control siRNA and siRNA for either prostasin or fibronectin in 6-well plates for 72 hours. The cDNA was prepared from 2 μg of total RNA using the High-Capacity cDNA Reverse Transcription kit (Applied Biosystems, Waltham, MA). Gene expression analyses were performed using the TaqMan^®^ Individual Gene Expression assays for human PRSS8 (Hs00173606, Applied Biosystems) and FN1 (Hs01549976, Applied Biosystems). Assays were conducted on at least three biological replicates using the TaqMan^®^ Fast Universal PCR Master Mix and 50 ng of cDNA/well, and all reactions were run on an Applied Biosystems StepOnePlus^™^ system. All genes were normalized to the 18S ribosomal RNA (4332641, Applied Biosystems) and HPRT1 (4332657, Applied Biosystems).

### Statistics

Methods are in “Supplemental Materials and Methods”.

## Supplementary information

Supplementary figures

Supplementary materials and methods

Supplementary materials and methods

## Data Availability

Original qPCR and western blot data supporting this study’s findings are provided within the article and supplementary files. Further data are available from the corresponding author upon request.
