# Supplementary figures and images for: Metastasis suppressing properties of the cell-surface anchored serine protease prostasin: new functional and mechanistic insights from breast cancer
Source: Oncogenesis. 2026 Apr 17;15(1):24. doi: 10.1038/s41389-026-00615-3 (PMC13213059; doi:10.1038/s41389-026-00615-3)

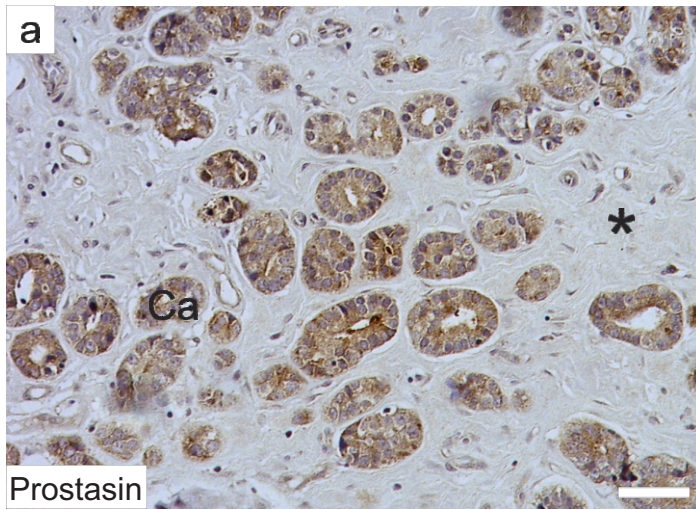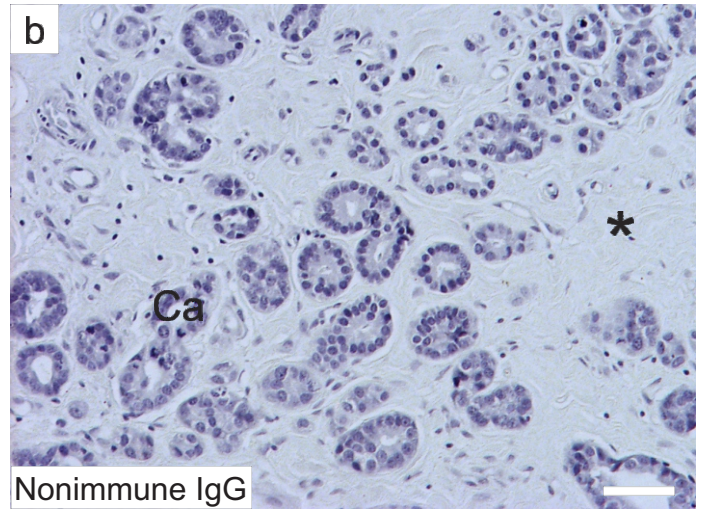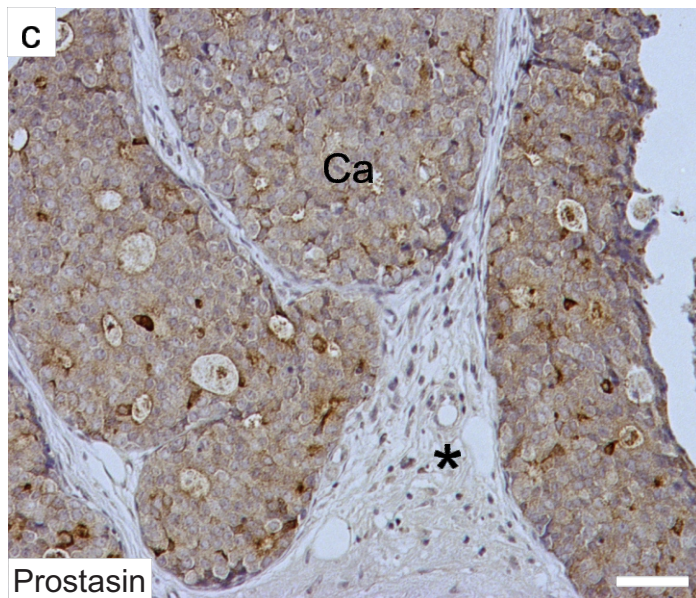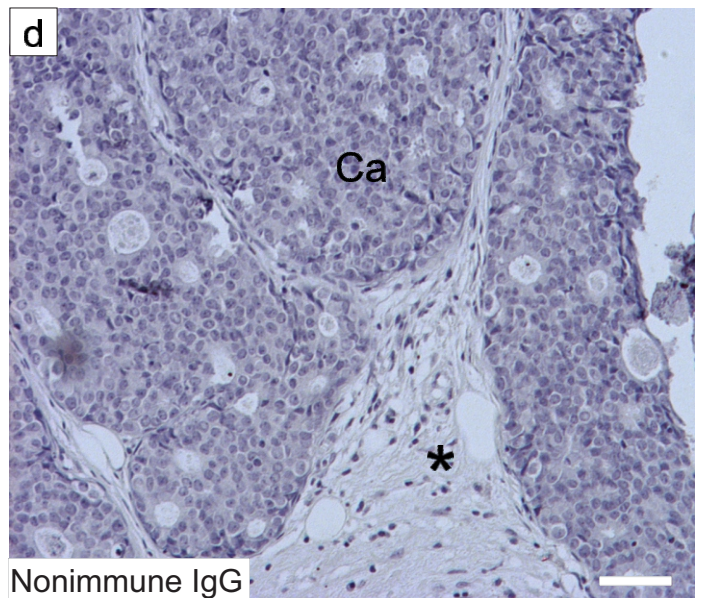

a

Mammary gland whole mount

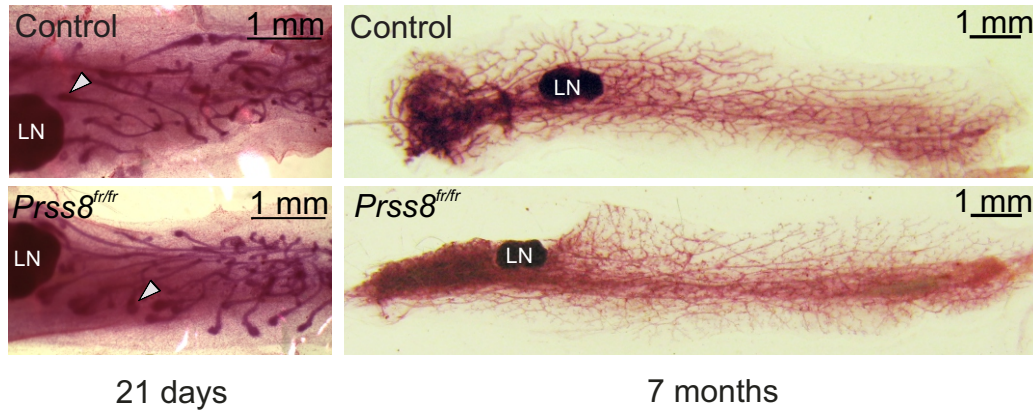

b

Offspring weight

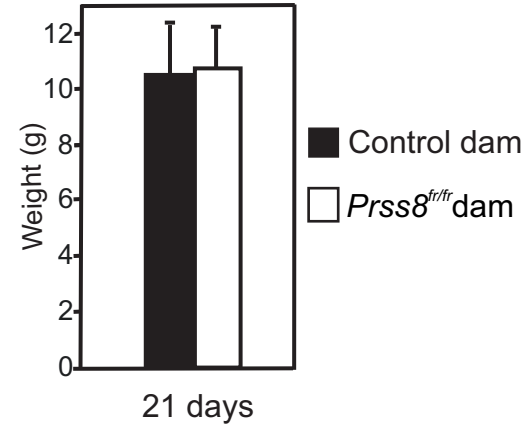

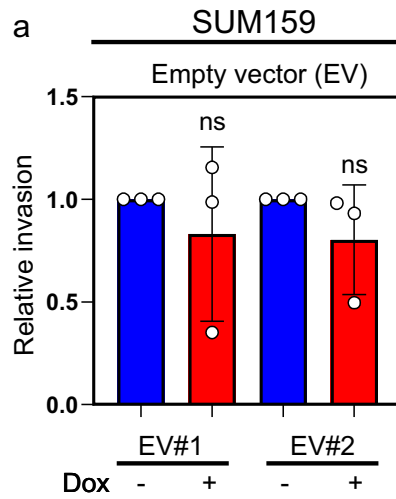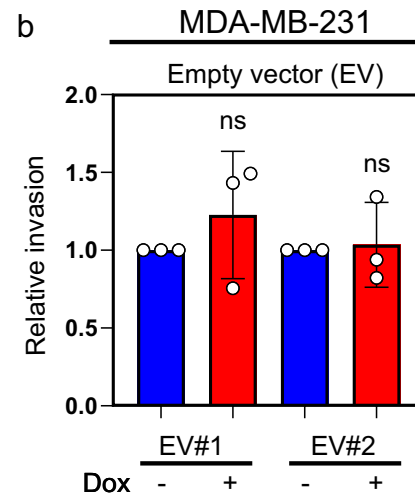

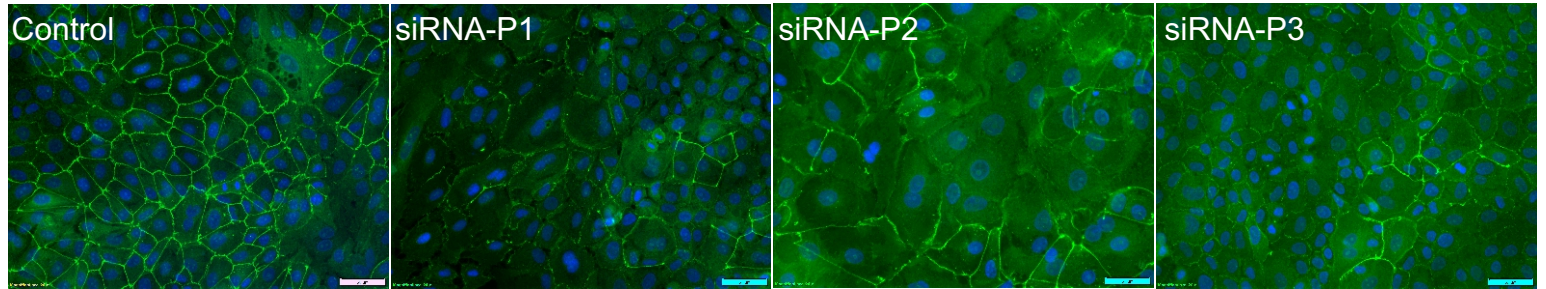

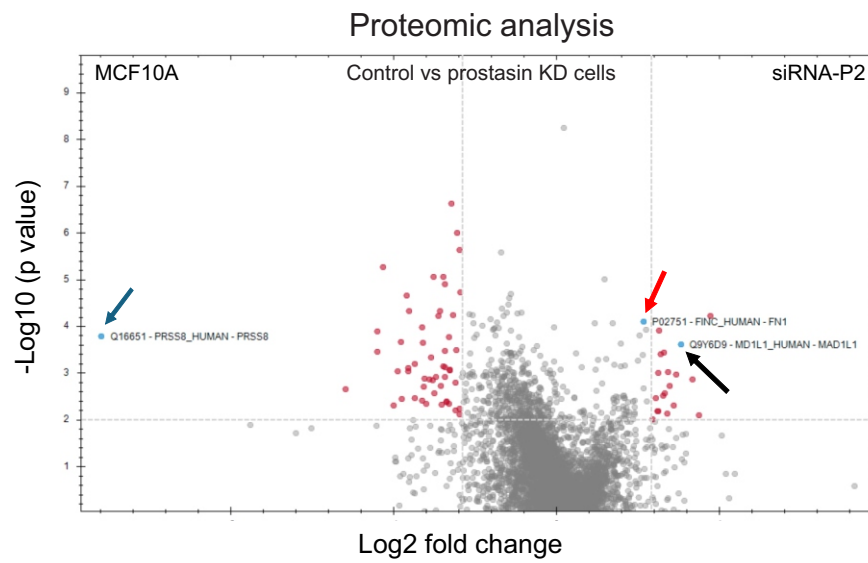

a

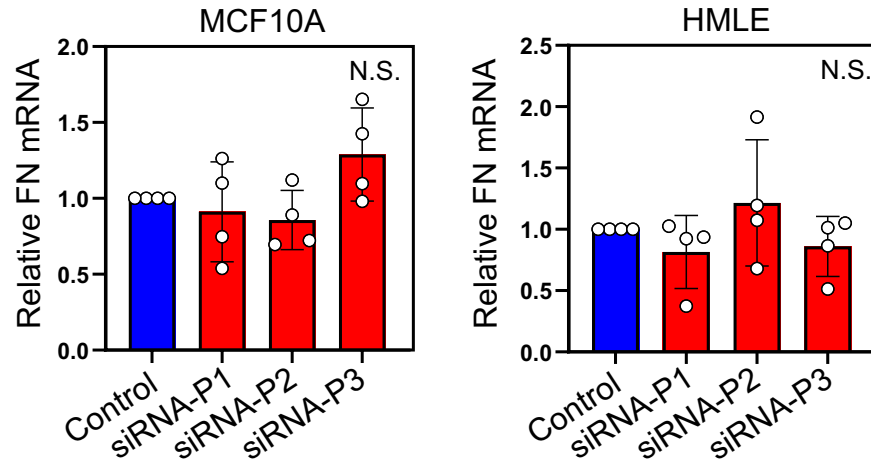

b

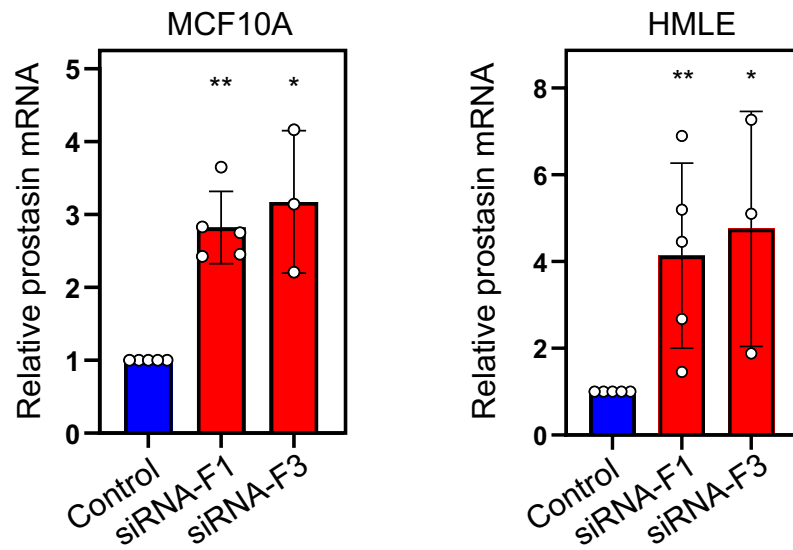

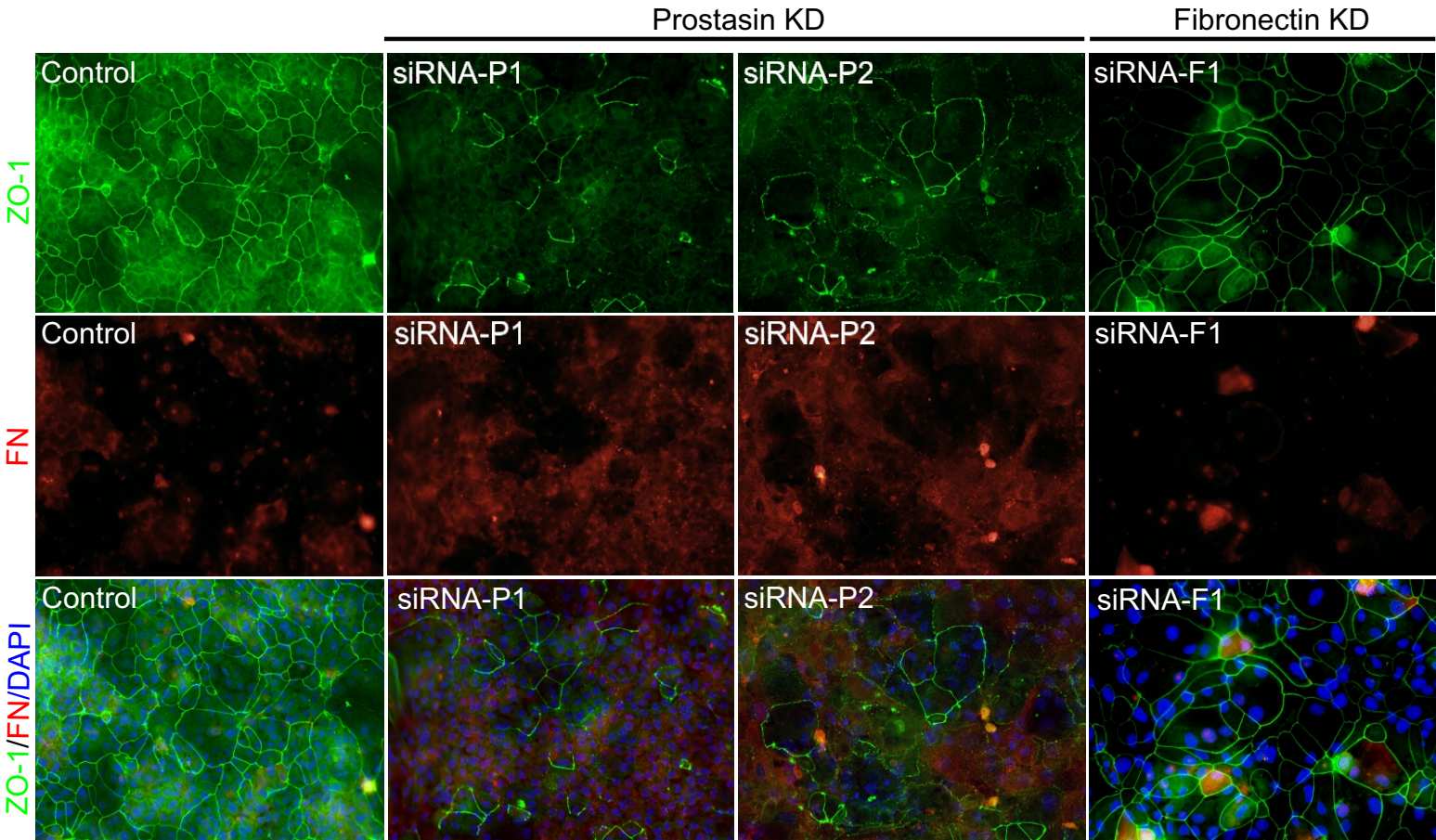

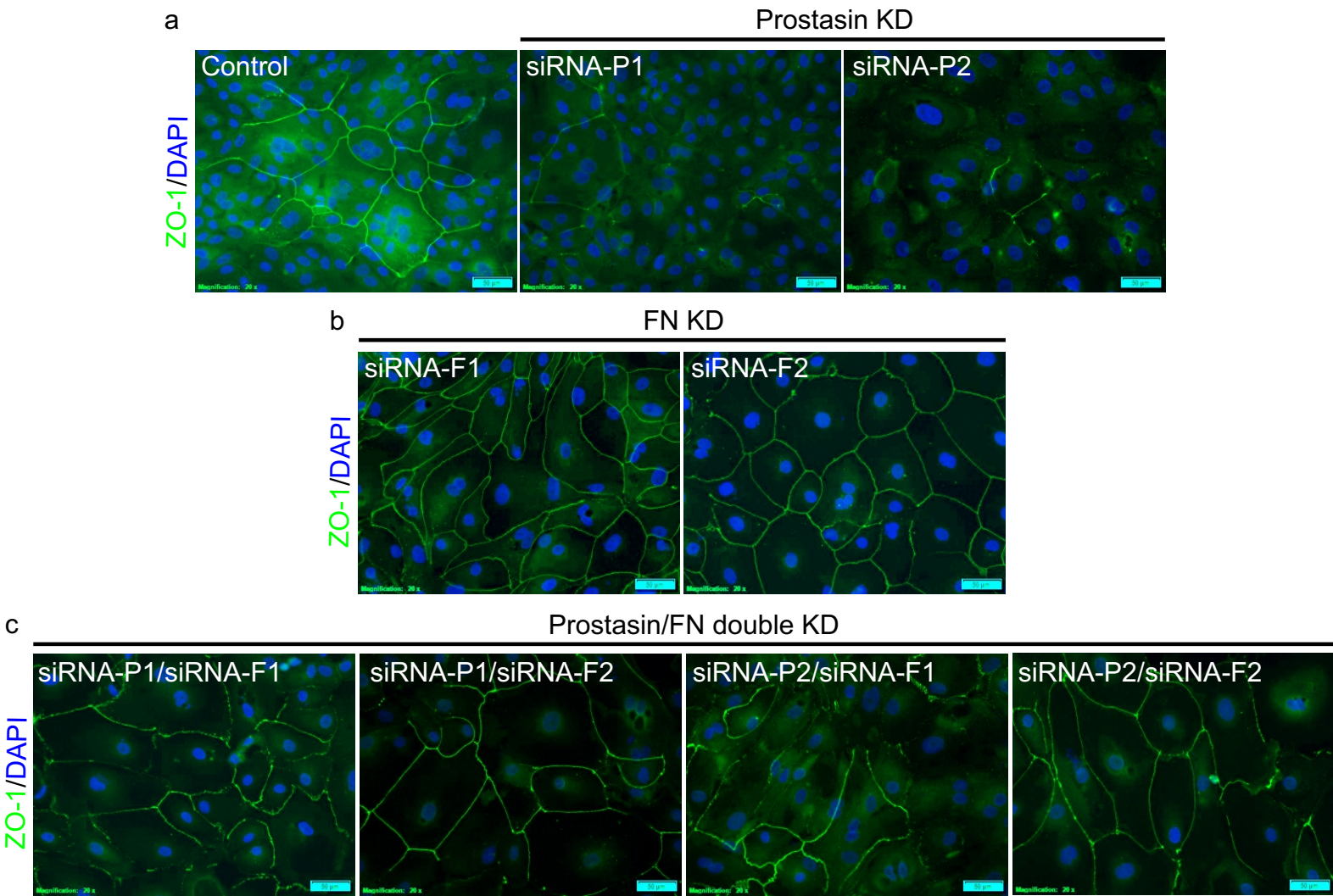

Supplement: Supplementary file 1 — Supplementary figures [file 41389_2026_615_MOESM1_ESM.pdf]
