# Supplementary material for: Metastasis suppressing properties of the cell-surface anchored serine protease prostasin: new functional and mechanistic insights from breast cancer
Source: Oncogenesis. 2026 Apr 17;15(1):24. doi: 10.1038/s41389-026-00615-3 (PMC13213059; doi:10.1038/s41389-026-00615-3)
Supplement: Supplementary file 2 — Supplementary materials and methods [file 41389_2026_615_MOESM2_ESM.pdf]

Supplementary figure legends

Supplementary Fig. S1. Prostasin protein expression in invasive ductal carcinoma. Representative prostasin immunohistochemistry in human breast tissues using a mouse anti-prostasin antibody. **a-b** Expression of prostasin in serial sections from a grade 1 IDC using anti-prostasin **a** or non-immune isotype control IgGI **b**. **c-d** In grade 2 lesions, prostasin displays a mostly diffuse staining **c** with no detectable staining when the anti-prostasin antibody is replaced with isotype control IgGI **d**. Size bars=50 µm. Ca=carcinoma cells. Asterisk=stromal tissue.

Supplementary Fig. S2. Normal development and function of prostasin-deficient mammary glands. a Representative mammary gland whole mounts from control and *PRSS8*$^{fr/fr}$ female littermate mice at postnatal days 21 and 7 months. No difference in epithelial elongation, branching, or terminal end bud (arrow heads) formation was detected. Size bars=1 mm. **b** Pups from control (N=25) and *PRSS8*$^{fr/fr}$ (N=26) dams were weighed at weaning (21 days after birth). No significant difference in weight was observed, indicating normal lactation function in *PRSS8*$^{fr/fr}$ mammary glands. LN=Lymph Node

Supplementary Fig. S3. Reconstituted basement-membrane invasion assay. Two stable independent clones (EV1 and EV2) for each of the SUM159 **a** and MDA-MB-231 cell lines **b** transfected with the empty vector (EV) that was used for prostasin expression in Fig. 3 were tested in invasion assays. Cells were treated with dox (+, red bars) or vehicle (-, blue bars) for 72 hrs prior to invasion. Cells that invaded through the matrix were stained and counted. Data from 3 independent experiments in triplicate for each clone normalized to control conditions are shown. No significant difference. Unpaired two-tailed t-test used to determine statistical significance. Error bars represent S.D.

Supplementary Fig. S4. Prostasin silencing in human mammary epithelial cells disrupts tight junction integrity. MCF10A cells were transfected with control siRNA (scrambled %GC matched) or three non-overlapping prostasin siRNAs (siRNA-P1, P2, or P3) for 72 hours at the time of seeding. Cells were fixed, permeabilized, incubated overnight with anti-ZO-1 antibody, and analyzed by fluorescent microscopy. Nuclei (DAPI) (*blue*), ZO-1 (*green*). Disrupted ZO-1 cell surface staining is observed upon prostasin silencing. Representative of five independent experiments. A different independent experiment is shown in Fig.4a.

Supplementary Fig. S5. Discovery mass spectrometry. MS-based quantitative proteomic analysis comparing the proteomes of MCF10A cells transfected with non-targeting scrambled and siRNA-mediated knockdown of prostasin (siRNA-P2). Blue arrow=*PRSS8/*Prostasin (P=0.0002), red arrow=*FN1*/Fibronectin (P value=0.00008), black arrow=*MAD1L/* Mitotic Arrest Deficient 1 Like (P=0.0002). Data using siRNA-P1 for prostasin silencing is shown in Fig. 4c.

Supplementary Fig. S6. Prostasin and FN transcript quantification by RT-qPCR in human mammary epithelial cells. a MCF10A (left) and HMLE cells (right) were transiently transfected with control siRNA (non-targeting scrambled %CG matched) or siRNAs targeting prostasin (siRNA-P1, P2, or P3) for 72 hours. The relative abundance of fibronectin mRNA in prostasin silencing conditions relative to the matching control is shown. Minimum of N=3 independent experiments per condition. No differences in FN mRNA levels upon prostasin silencing were detected (N.S.) **b** MCF10A (left) and HMLE (right) cells were transiently transfected with control siRNA (non-targeting scrambled %CG matched) or siRNAs targeting fibronectin (siRNA-F1, F2, or F3) for 72 hours. The relative abundance of prostasin mRNA upon FN prostasin silencing relative to the matching control is shown. Minimum of N=3 experiments per condition (*P<0.05,

**P<0.01). Unpaired two-tailed t-test used to determine statistical significance. Error bars represent S.D.

Supplementary Fig. S7. Mutually exclusive expression of prostasin and fibronectin in human mammary epithelial cells. MCF10A cells were transiently transfected with scrambled control siRNA (left panels), siRNAs silencing prostasin (P1 and P2, middle panels), or FN (F1, right panels) for 72 hours at the time of seeding. Cells were fixed, permeabilized, incubated overnight with anti-ZO-1 (upper panels) and anti-fibronectin antibodies (middle panels) and analyzed by fluorescent microscopy. Nuclei (DAPI) (*blue*), ZO-1 (*green*), fibronectin (*red*). Merged ZO-1, FN, and DAPI, lower panels. Cells with strong cellular FN signal generally displayed low ZO-1 staining and in cells with minimal FN staining, cell-surface expression of ZO-1 was detected. Edited photos of siRNA-P1 and siRNA-P2 are shown in Fig. 5b. A different independent experiment is shown in Fig. 5a. Data representative of 5 independent experiments.

Supplementary Fig. S8. Concomitant silencing of FN and prostasin rescues the TJ defects caused by prostasin loss. MCF10A cells were transfected with control siRNA (scrambled %GC matched), **a** two non-overlapping prostasin siRNAs (siRNA-P1, P2), **b** two non-overlapping FN siRNAs (siRNA-F1, siRNA-F2), or **c** four different combinations of prostasin/FN siRNA. Cells were fixed, permeabilized, incubated overnight with anti-ZO-1 antibody, and analyzed by fluorescent microscopy. Nuclei (DAPI) (*blue*), ZO-1 (*green*). Disrupted ZO-1 cell surface staining is observed upon prostasin silencing **a**, whereas clear continuous ZO-1 staining is present upon FN silencing and prostasin/FN silencing **b,c**. Slides from this experiment were also used for ZO-1/FN co-staining shown in Fig. 5. Data representative of 5 independent experiments.
