# Supplementary material for: Metastasis suppressing properties of the cell-surface anchored serine protease prostasin: new functional and mechanistic insights from breast cancer
Source: Oncogenesis. 2026 Apr 17;15(1):24. doi: 10.1038/s41389-026-00615-3 (PMC13213059; doi:10.1038/s41389-026-00615-3)
Supplement: Supplementary file 3 — Supplementary materials and methods [file 41389_2026_615_MOESM3_ESM.pdf]

Animals

All procedures involving live animals were performed in an Association for Assessment and Accreditation of Laboratory Animal Care International-accredited vivarium following institutional guidelines and standard operating procedures. Mice carrying the frizzy *(fr)* allele have a missense mutation in the *Prss8* gene causing a V170D amino acid substitution. Frizzy mice ($Prss8^{fr/fr}$) were acquired from Jackson Laboratories (FS/EiJ stock # 000619, Bar Harbor, ME). Genotyping was performed using the following primer sets for the frizzy wildtype allele (PrHy_WT51) CCAACGGCCTTCACTGTACTGT and (PrHy_31) CCCTCCATGGGACAAGAGAGT and for the Frizzy mutated allele: (PrHy_mut51) TTTCCCAACGGCCTTCACTGTACTGA and (PrHy_31) CCCTCCATGGGACAAGAGAGT. Transgenic mice carrying the MMTV-Polyoma virus middle T oncogene (PyVT or PymT) in the FVB/N background (strain: FVB/N-Tg (MMTV-PyVT)634Mul) were obtained from Jackson Laboratories and crossed with $Prss8^{fr/fr}$ mice to generate F1 PymT/$Prss8^{+/fr}$ mice. Male F1 PymT/$Prss8^{+/fr}$ mice were then crossed with $Prss8^{fr/fr}$ mice to generate F2 study cohorts of PymT/ $Prss8^{+/fr}$ (hereafter referred to as $Prss8^{+}$, prostasin-sufficient mice, or control mice) and PymT/$Prss8^{fr/fr}$ (hereafter referred as $Prss8^{fr/fr}$ or prostasin-deficient mice) female littermates. Genotyping for the PymT allele was performed with the following primer sets: PymT up: (5'-CGGCGGAGCGAGGAACTGAGGAGAG-3'), and PymT down: (5'-TCAGAAGACTCGGCAGTCTTAGGCG-3'). Tumor onset, tumor size, tumor burden, and metastasis data were measured/recorded by an investigator unaware of the genotypes of individual mice. All experiments were littermate controlled.

Cell Culture

Human breast epithelial cells, MCF10A were cultured in Dulbecco's modified Eagle media and Ham's F12 (DMEM/F12, Gibco, Life Technologies, Grand Island, NY) supplemented with 5% Horse serum (Gibco, Life Technologies, Grand Island, NY), 10mM HEPES (Millipore Sigma, Burlington, MA), 1μg/mL hydrocortisone (Sigma-Aldrich, St. Louis, MO), 1X Insulin-Transferrin-Selenium (ThermoFisher, Waltham, MA), 10ng/mL EGF (Millipore Sigma, Burlington, MA), 10 units/mL Penicillin, and 10μg/mL streptomycin (Gibco, Life Technologies, Grand Island, NY).

Human Mammary Epithelial cells, HMLE, were cultured in DMEM/F12 supplemented with 10% fetal bovine serum (FBS) (R & D Systems, Minneapolis, MN), 10μg/mL insulin (Gibco, Life Technologies, Grand Island, NY) 0.5μg/mL hydrocortisone (Sigma-Aldrich, St. Louis, MO), 10ng/mL EGF (Millipore, Sigma Burlington, MA) 10units/mL Penicillin, and 10μg/mL streptomycin (Gibco, Life Technologies, Grand Island, NY).

Triple negative breast cancer cell line, SUM159, were cultured in Ham's F12K Medium supplemented with 10% tetracycline-free FBS (Takara Bio USA, San Jose, CA), 1μg/mL hydrocortisone (Sigma-Aldrich, St. Louis, MO), 5μg/mL insulin (Gibco, Life Technologies, Grand Island, NY), 10units/mL Penicillin, and 10μg/mL streptomycin (Gibco, Life Technologies, Grand Island, NY).

Triple negative breast cancer cell lines MDA-MB-468 and MDA-MB-231 (ATCC, Manassas, VA) were cultured in Dulbecco's Modified Eagle media (DMEM, Gibco, Life Technologies, Grand Island, NY) supplemented with 10% tetracycline-free FBS (Takara Bio USA, San Jose, CA) 10 units/mL Penicillin, and 10μg/mL streptomycin (Gibco, Life Technologies, Grand Island, NY).

Human embryonic kidney HEK293FT (Thermo Fisher, Waltham, MA) cells used for lentivirus production were cultured in DMEM (Gibco, Life Technologies, Grand Island, NY) containing 10% FBS (R & D Systems, Minneapolis, MN). Malignant MCF-7 cells (ER+/PR+, HER2-) were cultured in Dulbecco's modified Eagle's media (DMEM) supplemented with (Gibco, Life Technologies, Grand Island, NY) supplemented with 10% FBS (R & D Systems, Minneapolis, MN). Malignant triple negative (ER-/PR-, HER2-) BT-20 cells were grown in Eagles + NEAA media (Eagle's MEM supplemented with phenol red, with 2 mM L-glutamine and Earle's BSS adjusted to contain 1.5 g/L sodium bicarbonate, 0.1 mM non-essential amino acids, 1mM sodium pyruvate, and 10% FBS). Malignant triple negative HCC1937 cells and ER-/PR-, HER2+ HCC1954 cells were grown in phenol-red containing RPMI + L-GLUT media (RPMI-1640 media with 2 mM L-glutamine adjusted to contain 1.5 g/L sodium bicarbonate, 4.5 g/L glucose, 10 mM HEPES, 1 mM sodium pyruvate, and 10% FBS). All cell lines were regularly tested to ensure they were free from mycoplasma.

Immunohistochemistry - human breast cancer tissues arrays

Tissue arrays (BR803, BR2083, BR2085, BR2086) were from US Biomax, Inc. (Derwood, MD). The arrays were deparaffinized with xylene and hydrated with graded ethanol solutions. Antigen retrieval was performed using heated citrate buffer, at reduced pH (Bethyl Laboratories, Montgomery, TX) and endogenous peroxidases were quenched by incubating the slides in 3% H_2O_2. The arrays were blocked with 2.5% horse serum albumin (Vector Laboratories, Burlingame, CA) in PBS and incubated overnight at 4 °C with 5 µg/ml mouse anti-human prostasin (612173, BD Biosciences, San Jose, CA) or rabbit anti-human prostasin (HPA030436, Millipore Sigma, Burlington, MA) in a humidity chamber. All washing steps were performed using PBS. As negative

controls, non-immune rabbit IgG (5 μg/ml) (12-370, Millipore Sigma, Burlington, MA) or mouse IgG2a (E5Y6Q, Cell Signaling, Danvers, MA) were used. Bound antibodies were visualized using ImmPRESS HRP Horse Anti-Mouse IgG Polymer Kit, Peroxidase (MP-7402-15, Vector Laboratories, Newark, CA) or ImmPRESS HRP Goat Anti-Rabbit IgG Polymer Kit, Peroxidase (MP-7451-15, Vector Laboratories, Newark, CA). 3,3'-Diaminobenzidine (DAB) was used as the substrate (Millipore Sigma, Burlington, MA) and the arrays were counterstained with *hematoxylin* QS (Vector Laboratories, Newark, CA). All microscopic images were acquired on a Zeiss Scope A.1 using digital imaging.

Assessment of staining intensities was performed by manual evaluation by an investigator unaware of the tumor grades. Scores were assigned on the basis of the intensity and extent of epithelial cell/cancer cell staining in 20x microscopic fields using an arbitrary scale from zero to four where 0 = no epithelial cells stained, 1= many epithelial cells stained weakly OR a few stained moderately with the majority unstained, 2 = the majority of epithelial cells stained weakly OR many epithelial cells stained moderately OR few epithelial cells stained strongly with the majority stained weakly or not at all, 3= many epithelial cells stained strongly OR the majority stained moderately, and 4= the majority of epithelial cells stained strongly.

Immunohistochemistry – mouse tissues

Tissues were fixed for 24 hours in 10% neutral-buffered zinc formalin (Z-fix, Anatech, Battle Creek, MI), processed into paraffin, and sectioned. Antigens were retrieved by heating in an epitope retrieval buffer with reduced pH (Bethyl Laboratories, Montgomery, TX). Prostasin was detected with 1μg/ml mouse anti-human prostasin (612173, BD Biosciences, San Jose, CA) using the M.O.M. (Mouse on Mouse) Elite® Immunodetection Kit, Peroxidase (PK-2200, Vector Laboratories, Newark, CA) according to manufacturer's instructions. 3,3'- DAB was used as the

substrate (Millipore Sigma, Burlington, MA), and slides were counterstained with *hematoxylin* QS (Vector Laboratories, Newark, CA). Cell proliferation was visualized by intraperitoneal injection of 100 µg/g BrdU (Millipore Sigma, Burlington, MA) 2 h before euthanasia. BrdU incorporation was detected with a rat anti-BrdU antibody (Accurate Chemical and Scientific Corporation). All microscopic images were acquired on a Zeiss Scope A.1 using digital imaging. Assessment of staining intensities was performed by evaluation by an investigator unaware of the tumor grades.

Immunocytochemistry

MCF10A and HMLE cells were reverse-transfected with siRNA in 6-well tissue culture plates with coverslips. Cells were transiently transfected and, at 72 hours post-transfection, had the media removed and were washed in PBS three times. SUM159 prostasin WT and MDA-MB-231 prostasin WT cells were plated in 6-well tissue culture plates containing coverslips and treated with 100 ng/mL doxycycline (Sigma-Aldrich, St. Louis, MO) for 72 hours to induce prostasin expression. Following the prostasin induction, the media was removed, and the cells were washed three times in PBS. Cells on the coverslips were fixed in zinc formalin (Z-fix, Anatech, Battle Creek, MI) for 15 min at room temperature and then washed three times in PBS. For permeabilization, cells were treated with 0.05% Triton X-100 in PBS for 15 min on ice, then washed in PBS three times. Cells were then blocked in 5% BSA in PBS for 1 h before the addition of primary antibodies. Following the blocking, the coverslips were incubated with primary antibodies (1:100 rabbit anti-ZO-1 (D6L1E) Alexa Fluor® 488, Cell Signaling, Danvers, MA; 1:100 rabbit anti-FN1 (E5H6X) Alexa Fluor® 488 Cell Signaling, Danvers, MA; 10 µg/mL sheep anti-fibronectin (AF1918) R & D Systems Minneapolis, MN; 1:200 rabbit anti-prostasin (PA5-80945) ThermoFisher, Waltham, MA) overnight at 4 °C. Following the primary antibody incubation, the coverslips were washed three times in PBS. Unconjugated sheep anti-fibronectin

coverslips were incubated with 1:1000 donkey anti-sheep secondary AlexaFluor ® 555 (A-21436 ThermoFisher, Waltham, MA), and unconjugated rabbit anti-prostasin coverslips were incubated with 1:1000 goat anti-rabbit secondary AlexaFluor® 488 (A32731, ThermoFisher, Waltham, MA) on ice for 3 hours. After washing with PBS three times, coverslips with cells were mounted with ProLong Diamond Antifade Mountant with DAPI (Invitrogen). Fluorescence images taken with an Olympus BX53 (20X) and confocal images were acquired on the Leica SP5 scope (63X) at the Microscopy Imaging and Cytometry Resources Core at Wayne State University School of Medicine. Acquired images were edited and merged using ImageJ software.

Invasion Assays

SUM159 and MDA-MB-231 WT and EV prostasin cells were first seeded in 6-well tissue culture plates in full serum media treated with (+) or without (-) 100ng/mL doxycycline (Sigma-Aldrich, St. Louis, MO) for 48 hours. Following treatment, 30,000 cells were seeded in triplicate onto transwell inserts (8.0 µM pore size, Corning, Corning, NY) pre-treated with Cultrex Basement Membrane Extract (R & D Systems, Minneapolis, MN) at 1 mg/mL in serum-free media. Inserts were placed in 24-well plates with the bottom chamber containing serum-supplemented media as a chemoattractant, and cells were cultured on inserts for 16 hours, after which invading cells were fixed and stained using Kwik-Diff (Siemens, Deerfield, IL). Images of inserts were acquired using an EZ4D Stereo Zoom microscope with a digital camera (Leica Microsystems, Buffalo Grove, IL). Invaded cells were quantified using ImageJ software.

Western Blot

Cultured human cells were washed three times with ice-cold PBS and lysed in-well using ice-cold RIPA buffer (150 mM NaCl; 50 mM Tris/HCl, pH 7.4, 0.1% SDS; 1% NP-40) with protease

inhibitor cocktail (Sigma-Aldrich, St. Louis, MO) and phosphatase inhibitor cocktail (Sigma Aldrich, St. Louis. MO), and cleared by centrifugation at 16,000g at 4 °C. Protein concentrations were determined using a Pierce BCA Protein Assay Kit (Thermo Fisher Scientific. Waltham, MA). Proteins were separated by SDS-PAGE under reducing conditions using 10% Mini-Protean gels or Criterion TGX midi gels (Bio-Rad, Hercules, CA) and transferred onto PVDF membranes. Membranes were blocked with 5% (w/v) dry milk powder or 5% BSA in TBS-T (Tris-buffered saline, 0.1% Tween-20) for 1 h at room temperature and subsequently incubated overnight at 4 °C in primary antibodies diluted in 5% dry milk powder/TBS-T or 5% BSA/TBS-T. Primary antibodies used for western blotting include 1:250 mouse anti-human prostasin (mAb) (612173, BD Biosciences, San Jose, CA), 1:1000 mouse anti-ß-Actin (NB600-501, Novus Biologicals, Centennial, CO), 1:1000 rabbit anti-fibronectin (E5H6X, Cell Signaling, Danvers, MA), and 1:1000 rabbit anti-ZO-1 (D6L1E, Cell Signaling, Danvers, MA).

RNAi-mediated gene silencing

Transient knockdown of prostasin and/or fibronectin in MCF10A and HMLE cells was performed using Lipofectamine RNAiMAX according to the manufacturer’s instructions (Invitrogen, Life Technologies, Inc, Waltham, MA) with % GC-matched negative controls. Stealth siRNA duplexes targeting prostasin and fibronectin were obtained from Invitrogen Life Technologies (HSS108631 corresponding to prostasin siRNA-1, HSS108633 corresponding to prostasin siRNA-2, and HSS183514 corresponding to prostasin siRNA-3; HSS103780 corresponding to fibronectin siRNA-1, HSS103782 corresponding to fibronectin siRNA-2, and HSS177362 corresponding to fibronectin siRNA-3). Transfections were performed with 1.5µL of 20 µM siRNA in a 6-well plate format in complete cell media in the absence of penicillin/streptomycin.

Lentiviral Transfection

The ViraPower™ T-REx™ Lentivirus Expression System (Invitrogen, Waltham, MA) was used for the generation of replication-incompetent lentivirus stably expressing a recombinant human prostasin under doxycycline regulation. Plasmids of pENTR-Pro harboring a human prostasin complementary DNA (cDNA) were created via a directional TOPO cloning of the PCR product containing the appropriate prostasin cDNA into the pENTR™/D-TOPO® vector. The lentiviral prostasin expression construct was generated by an LR recombination between the entry clone and pLenti4/TO/V5-DEST vector [54]. To generate the empty vector (EV) plasmid, the WT prostasin plasmid was treated with *EcoR I* restriction enzyme (New England Biolabs, Ipswich, MA) to remove the prostasin DNA, followed by a self-ligation. The final expression constructs were confirmed by Sanger sequencing. Lentivirus was produced in the HEK293FT (ATCC, Manassas, VA) cells according to the manufacturer's instructions using the ViraPower™ Bsd Lentiviral Support Kit (Thermo Fisher, Waltham, MA). SUM159 and MDA-MB-231 cells were transduced with pLenti CMW TetR Blast (TR) [55] (Addgene, Watertown, MA) and selected using 5 µg/mL Blasticidin (InvivoGen, San Diego, CA). The SUM159TR and the MDA-MB-231TR cells resistant to Blasticidin selection were expanded and transduced with the EV or WT prostasin and selected using 100 µg/mL Zeocin® (Gibco, Life Technologies, Grand Island, NY). SUM159TR EV and WT prostasin cells and MDA-MB-231TR EV and WT prostasin cells were grown in the presence of both Zeocin® and Blasticidin and were serially diluted in 96 well plates to form single colonies for 2 weeks. Single cell colonies were expanded in 24-well and 12-well plates and tested for the induction of prostasin using 100 ng/mL doxycycline versus the vehicle treated cells from the same colony.

Mass Spectrometry Sample Preparation and Analysis

Cell pellets were solubilized in 200 µL of 2.5% Lithium dodecyl sulfate detergent (LiDS) then heated to 95°C for 5 minutes prior to filtration through spin columns (Pierce #89868, Thermo Fisher, Waltham, MA) to produce a clear lysate solution. An aliquot of each filtered lysate was taken for BCA protein analysis and the remainder of each sample was buffered with 40 mM tetraethylammonium bicarbonate (TEAB), (60-044-974, Fisher Scientific, Waltham, MA) then reduced with 5 mM dithiothreitol (DTT) and alkylated with 15 mM iodoacetamide (IAA) with excess IAA quenched after a 30 minute incubation by the addition of a second aliquot of 5 mM DTT. Samples were acidified by addition of 20 µL of 12% phosphoric acid then proteins were precipitated by addition of 1 ml of 90% MeOH in 100 mM TEAB. Pellets from the precipitation were washed with 0.5 ml of 80% MeOH in 10 mM TEAB. Washed precipitates were dried on the bench then resuspended in 250 ul of 100 mM NaCl, 1 mM $CaCl_2$, 40 mM TEAB and 0.5% deoxycholate (DOC). Aliquots of 30 µg protein were taken from each sample and 1.0 µg trypsin (V5113, Promega, Madison, WI) was added, followed by incubation overnight at 37°C to complete the digestion. LC-MS/MS analysis was performed using a Thermo scientific Vanquish-Neo chromatography system with an Acclaim PepMap 100 trap column (100 µm × 2 cm, C18, 5 µm, 100Å, Thermo Fisher, Waltham, MA), and Thermo Scientific Easy-Spray PepMap RSLC C18 75 µm x 25 cm column (Thermo Fisher, Waltham, MA). A gradient starting at 2% of a solution containing 80% acetonitrile with 0.1% formic acid and finishing at 42% acetonitrile 120 minutes later is used for all samples. Data independent analysis was performed on an Orbitrap Eclipse MS system (Thermo Fisher, Waltham,MA). MS1 spectra were acquired at 120,000 resolution in the 400 to 1000 Da mass range with an AGC of 3e6. MS2 spectra were acquired in the Orbitrap and collected at 15,000 resolution. Fragmentation MS2 spectra were collected using 15 Da windows

over the range 400 to 1,000 Da with HCD fragmentation at a collision energy of 30, a maximum injection time of 120 msec, and an AGC target of 3e6.

Protein identification and quantification

Mass spectrometry data were processed with Spectronaut 19.0 (Biognosys, Schlieren, Switzerland), using the directDIA strategy and the Pulsar search engine. Spectra were searched against the Human Uniprot FASTA database downloaded March 30, 2021. The search parameters included trypsin with up to two missed cleavages. Variable modifications are oxidation of M; and of protein N-termini by Acetylation, Met Loss or both Acetylation and Met loss. Carbamidomethylation of cysteine was a fixed modification. Quantification was accomplished using the MS2 data with default Spectronaut 19 settings. For the entire data set, the false discovery rate (FDR) was calculated using a cut-off of 1% for identification of precursors, peptides and protein groups.

TaqMan RT-PCR analyses

MCF10A and HMLE cells were reverse transfected with %GC matched control siRNA and siRNA for either prostasin or fibronectin in 6-well plates for 72 hours. The cDNA was prepared from 2 μg of total RNA using the High-Capacity cDNA Reverse Transcription kit (Applied Biosystems, Waltham, MA). Gene expression analyses were performed using the TaqMan® Individual Gene Expression assays for human PRSS8 (Hs00173606, Applied Biosystems, Waltham, MA) and FN1 (Hs01549976, Applied Biosystems, Waltham, MA). Assays were conducted on at least three biological replicates using the TaqMan® Fast Universal PCR Master Mix and 50 ng of cDNA/well, and all reactions were run on an Applied Biosystems StepOnePlus™ system. All genes were

normalized to the 18S ribosomal RNA (4332641, Applied Biosystems, Waltham, MA) and HPRT1 (4332657, Applied Biosystems, Waltham, MA).

Statistics

Sample sizes were chosen according to power analysis and best practices to ensure sufficient sample numbers for rigor and sound statistical analysis. Mouse cohort numbers were determined using power analysis to ensure studies have sufficient statistical power to detect a meaningful effect, while adhering to the 3Rs principles (Replacement, Reduction, Refinement). In the genetic cohorts, all mice were used to achieve a littermate-controlled study. The need to control for litter-to-litter variability and ensure animal welfare takes precedence over simple random assignment. Appropriate statistical tests were used to determine statistical significance and data meet the assumptions of the tests. All sample sizes were chosen to ensure experimental rigor and sufficient statistical power. All statistical analyses were performed using GraphPad Prism software. For immunohistochemical staining, differences in staining scores between the normal and the cancer grade groups were analyzed using the non-parametric Kruskal–Wallis ANOVA test with posthoc comparisons performed using the Dunn's test. Mann-U two-tailed tests were performed for the statistical analyses of final tumor burden, the Mantel-Cox log-rank test was performed to assess differences in tumor latency, and a chi-square test was performed to assess differences in lung metastatic outcomes incidence. Statistical tests to analyze differences in proliferation and apoptosis were done using two-tailed unpaired Student t-tests. For prostasin and fibronectin protein and RNA levels, the knockdown experiments were compared to the matching controls using an unpaired two-tailed t-test. For cell invasion assays, cells without doxycycline were used as controls compared to cells treated with doxycycline from the same experiment. Unpaired two-tailed t-tests were used to determine the statistical significance.
